# FBXW7β loss-of-function enhances FASN-mediated lipogenesis and promotes colorectal cancer growth

**DOI:** 10.1038/s41392-023-01405-8

**Published:** 2023-05-19

**Authors:** Wenxia Wei, Baifu Qin, Weijie Wen, Boyu Zhang, Haidan Luo, Yuzhi Wang, Hui Xu, Xiaoshan Xie, Sicheng Liu, Xin Jiang, Mengan Wang, Qin Tang, Jiayu Zhang, Runxiang Yang, Zongmin Fan, Haiwen Lyu, Junzhong Lin, Kai Li, Mong-Hong Lee

**Affiliations:** 1 Guangdong Provincial Key laboratory of Colorectal and Pelvic Floor Disease, The Sixth Affiliated Hospital, Sun Yat-sen University, Guangzhou, 510655 China; 2 Guangdong Institute of Gastroenterology, Guangzhou, 510655 China; 3 Second Department of Medical Oncology, The Third Affiliated Hospital of Kunming Medical University, Kunming, 650118 China; 4 Guangzhou Institute of Pediatrics, Guangzhou Women and Children’s Medical Center, Guangzhou Medical University, Guangzhou, 510623 China; 5 Department of Colorectal Surgery, Cancer Center, Sun Yat-sen University, Guangzhou, 510060 China; 6 Department of Oncology, The Sixth Affiliated Hospital, Sun Yat-sen University, Guangzhou, 510655 China

**Keywords:** Epigenetics, Cancer metabolism

## Abstract

Continuous de novo fatty acid synthesis is required for the biosynthetic demands of tumor. FBXW7 is a highly mutated gene in CRC, but its biological functions in cancer are not fully characterized. Here, we report that FBXW7β, a FBXW7 isoform located in the cytoplasm and frequently mutated in CRC, is an E3 ligase of fatty acid synthase (FASN). Cancer-specific FBXW7β mutations that could not degrade FASN can lead to sustained lipogenesis in CRC. COP9 signalosome subunit 6 (CSN6), an oncogenic marker of CRC, increases lipogenesis via interacting with and stabilizing FASN. Mechanistic studies show that CSN6 associates with both FBXW7β and FASN, and antagonizes FBXW7β’s activity by enhancing FBXW7β autoubiquitination and degradation, which in turn prevents FBXW7β-mediated FASN ubiquitination and degradation, thereby regulating lipogenesis positively. Both CSN6 and FASN are positively correlated in CRC, and CSN6-FASN axis, regulated by EGF, is responsible for poor prognosis of CRC. The EGF-CSN6-FASN axis promotes tumor growth and implies a treatment strategy of combination of orlistat and cetuximab. Patient-derived xenograft experiments prove the effectiveness of employing orlistat and cetuximab combination in suppressing tumor growth for CSN6/FASN-high CRC. Thus, CSN6-FASN axis reprograms lipogenesis to promote tumor growth and is a target for cancer intervening strategy in CRC.

## Introduction

Rewiring of metabolic programs is often observed in cancer cells. There are at least three features of cancer cell metabolic deregulation: increased glycolysis and glutaminolysis, and increased de novo lipogenesis. The de novo synthesis of fatty acids is controlled by fatty acid synthase (FASN) using acetyl-CoA and malonyl-CoA. Fatty acids synthesis is a critical cellular process involved in energy storage, membrane biosynthesis, and generating signal mediators,^1^ which are critical for cancer development during tumorigenesis. Thus FASN overexpression is frequently observed in many cancers. However, the mechanisms behind FASN overexpression/activity regulation remain largely unclear. And FASN becomes an appealing cancer therapeutic target.^2–4^ FASN inhibition has impacts on tumorigenesis. For examples, *Fasn* knockdown leads to accumulation of malonyl-CoA, thereby causing lysine 1218 malonylation on mTOR and impeding mTOR complex 1 kinase activity.^5^ *Fasn* knockout delays hepatocarcinogenesis driven by sgPten/c-Met-driven in mice.^6^ Inhibition of fatty acid or sphingolipid synthesis leads to suppression of tumor development.^7^ But the applications to control FASN for cancer therapeutic efficacy remains largely unexplored.

The mammalian constitutive photomorphogenesis 9 (COP9) signalosome (CSN), a multi-subunit complex engaged in ubiquitination, is implicated in protein degradation, transcriptional regulation, signal transduction and tumorigenesis. It is interesting that all the 8 subunits of the mammalian COP9 shows sequence similarity compare with subunits of the “lid” complex of the 26S proteasome. Indeed, CSN functions as a scaffold that coordinates ubiquitination process and E3 ubiquitin ligase activity. For examples, CSN6 promotes degradation of some important E3 ligases like MDM2 and β-trcp to achieve biological effects.^8,9^ Emerging evidence shows that the CSN subunits is involved in cancer progression. Previous studies indicated that CSN6 gives rise to the colorectal malignancy, enhances CRC development and poor patient survival.^8^ CSN6 affects glycolysis, glutaminolysis, and SGOC amino acid metabolism.^10,11^ Its role in lipid metabolism has not been explored.

FBXW7 (F-box and WD repeat domain-containing 7) is a component of the multiprotein ubiquitin E3 ligase termed SCF (SKP1–CUL1–F-box protein) complex^12,13^ and is critical in substrate recognition. Many cancers have frequent FBXW7 mutations,^14–19^ and FBXW7 functions as a tumor suppressor. FBXW7 appears to be a target transcriptionally regulated by tumor suppressor p53.^20,21^ FBXW7 associates with specific proteins and enhances their ubiquitination and degradation.^22^ Most targeted substrates of FBXW7 are oncoproteins such as c-Myc,^23,24^ Cyclin E,^25^ c-JUN,^26,27^ MTOR,^28^ Notch,^29^ Aurora B,^30^ FOXO4, MCL-1^26,31–34^ and CHD6.^35^ The human FBXW7 gene encodes three isoforms (FBXW7α, FBXW7β, and FBXW7γ),^15,22^ and these three isoforms exhibit various subcellular localizations. FBXW7α is nucleoplasmic while FBXW7β is cytoplasmic, and FBXW7γ is predominately nucleolar. FBXW7 isoforms are generated from the alternative splicing of isoform-specific exons. The distinct subcellular localizations of the FBXW7 isoforms implied that they might target different substrates. For example, FBXW7β poorly interacts with c-Myc when compared with FBXW7α and FBXW7γ.^36^ All substrates of FBXW7 contain a consensus motif, so-called phospho-degron, also termed CPD (Cdc4 phospho-degron). Usually, the +4 position serine (primed phosphorylation) is firstly phosphorylated by GSK3 (glycogen synthase kinase 3) and then central phospho-threonine could be efficiently phosphorylated.^24,27,34,37–39^ The GSK-mediated phosphorylation of degron (TXXXS) generates a high-affinity binding site for FBXW7 and coordinates with FBXW7 targeting substrates degradation. FBXW7 is critical in cancer suppression. Nonetheless, FBXW7β and its associated targets, which are poorly defined in cancer study, still remain to be characterized.

The molecular alterations in CRC have been characterized extensively and provide routine clinical guide in determining CRC treatment. For example, frequent *RAS/BRAF* mutations impact on the clinical management of CRC, and it is documented that human colon cancers bearing mutated *KRAS* will not be responding to cetuximab (anti-EGFR monoclonal antibody) treatment. WT *KRAS* status is recommended for cetuximab treatment. However, CRC patients bearing WT *KRAS* respond to cetuximab only to some degrees.^40^ Thus, a more detailed study of deregulated EGF pathway in CRC needs to be further characterized to help guide treatment and improve clinical care.

In this study, we show that FASN is highly overexpressed in CRC. We characterize the critical regulators of the FASN overexpression in tumor development, including EGF, CSN6, GSK3β, and E3 ligase FBXW7β. CSN6 antagonizes cytosolic FBXW7β’s activity by enhancing FBXW7β autoubiquitination and degradation, thereby preventing FBXW7β/GSK3β-mediated FASN ubiquitination and degradation, lending support to why both CSN6 and FASN are positively correlated in cancer. The aberrant overexpression of CSN6-FASN axis in CRC leads to the metabolic reprogramming of lipid biosynthesis that instigates tumor growth and organoid formation, and results in poor cancer prognosis. Furthermore, we show that the EGF pathway facilitates tumor-promoting metabolic programs by elevating the steady-state expression of CSN6/FASN in CRC cells. Our studies provide pivotal insight into the signaling regulation of the EGF-CSN6-GSK3β-FBXW7β axis in modulating ubiquitin-mediated FASN degradation and lipogenesis. Importantly, combination of FASN inhibitor and cetuximab can suppress the tumor growth of patient-derived xenografts that have CSN6/FASN overexpression. Our studies of the role of EGF/CSN6 in hindering FBXW7β-mediated FASN ubiquitination reveal rational therapy for cancer intervention.

## Results

### CSN6 reprograms lipogenesis to promote the growth of cancer cells and PDOs

**Fig. 1:**
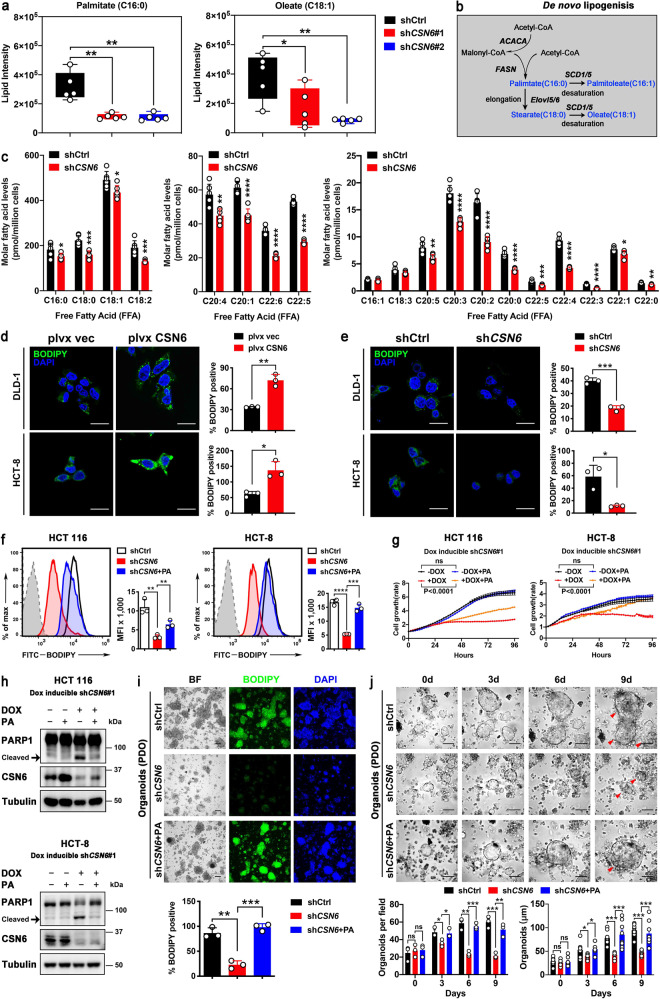
CSN6 is involved in de novo lipogenesis to promote the growth of CRC cell lines and patient-derived organoids. **a** Knockdown of CSN6 reduces palmitate (C16:0) and oleate (C18:1) production. Untargeted lipidomic analysis was performed in HCT 116 cells (*n* = 5 per group) infected with control shRNA or CSN6 shRNA. **b** Schematic overview of de novo fatty acid synthesis. *ACACA*, acetyl-CoA carboxylase; *FASN*, fatty acid synthase; *SCD1/5*, stearoyl-CoA desaturase 1 and 5; *Elovl5/6*, elongation of very long chain fatty acids protein 5 and 6. **c** Quantification of individual high-abundance (left), medium-abundance (middle) or low-abundance (right) free fatty acids in HCT 116 cells (*n* = 6 per group) infected with control shRNA or CSN6 shRNA. **d**, **e** Confocal microscopy images of intracellular lipid droplets in DLD-1 (upper) and HCT-8 (bottom) cells stained with BODIPY 493/503 dye (green). Nuclei were stained with DAPI (blue). Scale bar, 20 μm. Fluorescent BODIPY quantification was shown. **f** The effect of palmitate on CSN6-mediated lipogenesis was determined by BODIPY 493/503 staining followed by flow cytometry analysis. Representative histograms and mean fluorescence intensities (MFI) in HCT 116 (left) and HCT-8 (right) cells were shown. **g** Cell growth assay for dox inducible sh*CSN6#1* HCT 116 (left) and HCT-8 (right) cells pretreated with or without doxycycline (DOX, 200 μg/mL) for 48 h and then treated with ±200 μM exogenous palmitate (PA) in medium containing regular FBS. **h** Western blot analysis for cleaved poly (ADP-ribose) polymerase 1 (PARP1) expression in HCT 116 (top) and HCT-8 (button) cells with indicated treatment. Arrow denotes molecular weight of cleaved PARP1. **i** Fluorescence microscopy analysis of patient-derived organoids (PDOs) stained with BODIPY 493/503 dye (green) and DAPI (blue). Representative images and fluorescent BODIPY quantification of indicated treatment groups were shown. Scale bar, 100 μm. **j** Representative bright-field images and quantification of patient-derived organoids (PDO) in different time points were shown. All values are expressed as means ± SD. ns not significant, *P < 0.05, **P < 0.01, ***P < 0.001, ****P < 0.0001; as determined by two-way analysis of variance (ANOVA) (**g**), one-way ANOVA (**a**, **f**, **i**, **j**) or by two-sided Student’s *t*-test (**c**, **d**)

CSN6 is overexpressed in CRC.^8,10,41^ To investigate how CSN6 functions during CRC tumorigenesis, we explored its regulatory role in cancer hallmarks, focusing on metabolism reprogramming. We used mass spectrometry to perform untargeted lipidomic analyses in CSN6 knockdown (KD) cells. Lipidomic profiles were normalized to respective internal standards, and the result showed a significant downregulation of fatty acid synthesis, as demonstrated in lower amount of palmitate (C16:0) and oleate (C18:1) in CSN6 KD cells when compared with relative controls (Fig. 1a, b). Moreover, metabolite analysis of cells by LC-MS demonstrated a significant reduction in the pools of a series of free fatty acids (FFAs) after CSN6 KD, including the most predominant saturated fatty acids palmitate and stearate (Fig. 1b, c; Supplementary Fig. 1a–c). These results were further supported by intracellular staining with the lipophilic fluorescence dye BODIPY 493/503, indicating that CSN6 overexpression results in increased lipid stores, while CSN6 KD decreased the lipid contents in cells (Fig. 1d, e).

Consistent with this, flow cytometric studies also showed that CSN6 KD decreased the BODIPY staining when compared with related controls (Fig. 1f). Interestingly, CSN6 KD–mediated reduced intensity of BODIPY staining can be partially restored by the supplementation of palmitate, a key product of lipid synthesis (Fig. 1f). CSN6 KD led to cell growth inhibition (Fig. 1g; Supplementary Fig. 1d), apoptosis (Fig. 1h; Supplementary Fig. 1e, f), reduced colony formation (Supplementary Fig. 1g), and diminished cell migration (Supplementary Fig. 1h), and again palmitate attenuated these phenomena. We also generated patient-derived organoids (PDOs)^42,43^ from CRC patient specimens. CSN6 KD in PDOs reduced lipid accumulation (BODIPY staining) as well as organoid formation (number and size) (Fig. 1i, j). Under such a situation, palmitate supplementation can again rescue the lipid amount and alleviate PDO growth inhibition (Fig. 1i, j). These data suggest that CSN6 is critical in exerting lipid accumulating activity, thereby affecting tumor cell growth, cell apoptosis, and PDO growth.

### CSN6 associates with lipogenesis regulator FASN and attenuates ubiquitin-mediated degradation of FASN to enhance FASN protein stability

**Fig. 2:**
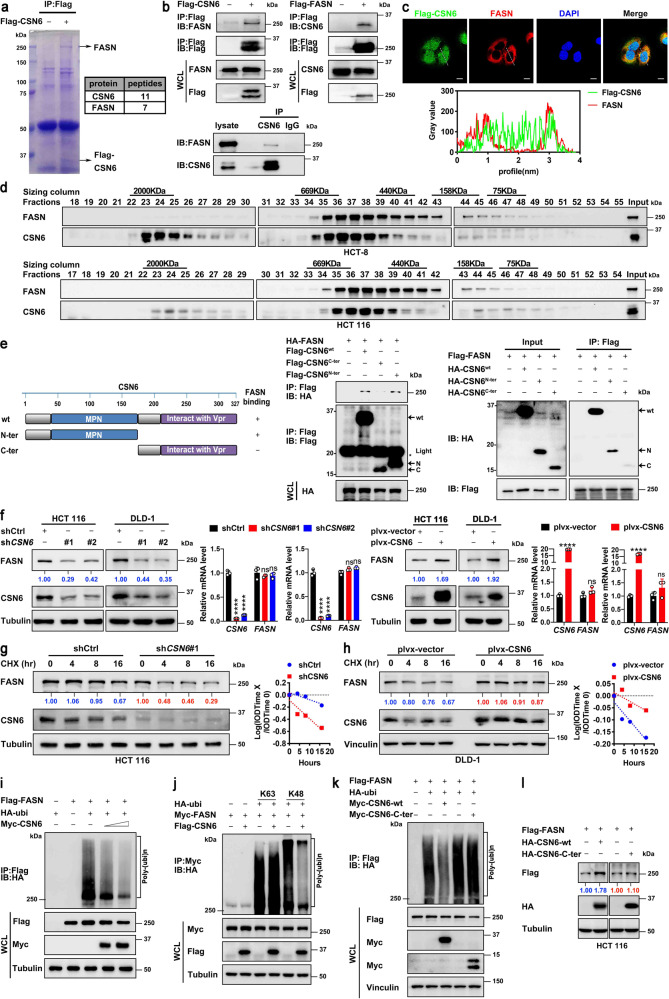
CSN6 interacts with FASN and attenuates ubiquitin-mediated degradation of FASN to enhance FASN protein stability. **a** Flag-CSN6 was transfected into DLD-1 cells. Cell lysates were immunoprecipitated with anti-Flag M2 agarose beads, and immunoprecipitates of anti-Flag-CSN6 were separated using SDS–PAGE and stained with Coomassie Brilliant Blue (left). The protein bands were retrieved and analyzed by mass spectrometry (right). The position of FASN and Flag-CSN6 are indicated by arrows. **b** Flag-CSN6 or Flag-FASN were transfected into HEK293T cells. Cell lysates were immunoprecipitated (IP) with anti-Flag M2 agarose beads and immunoblotted (IB) with the indicated antibodies (top). HCT 116 cell lysates were immunoprecipitated with anti-CSN6 antibodies followed by immunoblotting with the indicated antibodies (bottom). WCL, whole cell lysates. **c** Colocalization of FASN and CSN6 in cytoplasm of U2OS cells. U2OS cells expressing Flag-CSN6 were subjected to immunofluorescence analysis with anti-Flag-CSN6 (green) and anti-FASN (red) (top). Nuclei are stained with DAPI (blue). Scale bar, 10 μm. The colocalization coefficients between the indicated proteins were shown (bottom). **d** HCT-8 (top) and HCT 116 (bottom) cell proteins were extracted and fractionated by gel-filtration chromatography. Chromatographic eluant profiles and the eluted positions of proteins are shown. An equal volume from each chromatographic fraction was analyzed by western blotting with indicated antibodies. **e** CSN6 wild-type (wt), N-terminal (N-ter) or C-terminal (C-ter) was transfected into HEK293T cells. Cell lysates were immunoprecipitated with anti-Flag M2 agarose beads and immunoblotted with the indicated antibodies. Light, immunoglobulin light chain. **f** Western blot analysis of FASN protein in cells infected with indicated lentiviruses (left). qRT-PCR analysis was performed to measure the mRNA expression of *FASN* (right). **g**, **h** HCT 116 (**g**) or DLD-1 (**h**) cells infected with indicated lentiviruses were treated with cycloheximide (CHX, 100 μg/mL) for the indicated time points. Cell lysates were immunoblotted with indicated antibodies (left). The relative abundance of remaining FASN protein was normalized to the *t* = 0 controls (right). **i**–**k** HEK293T cells were transfected with indicated plasmids. MG132 (20 μM) was added to the cells 6 h before they were harvested. The ubiquitinated FASN proteins were pulled down with anti-Flag M2 agarose beads (**i**, **k**) or anti-myc beads (**j**) and detected with an anti-HA antibody. **l** HEK293T cells were transfected with the indicated HA-tag CSN6 constructs together with Flag-FASN. Cell lysates were immunoblotted with the indicated antibodies. All values are expressed as means ± SD. ns not significant, ****P < 0.0001; as determined by two-sided Student’s *t*-test or one-way ANOVA (**f**)

To investigate how CSN6 is involved in the reprogramming of lipid synthesis in CRC cells, we conducted mass spectrometry to identify CSN6-interacting partners linked to lipogenesis. Proteomics analysis showed that fatty acid synthase (FASN) was characterized to interact with CSN6 (Fig. 2a, Supplementary Table 1). Co-immunoprecipitation (co-IP) assay revealed that CSN6 could bind to endogenous FASN in HCT 116 cells (Fig. 2b). Semi-exogenous reciprocal interaction was also verified (Fig. 2b). Further, CSN6 co-localized with cytosolic protein FASN in the cytoplasm as indicated by immunofluorescence (IF) studies (Fig. 2c). To further support that CSN6 and FASN can form a complex in vivo, cellular proteins extracted from HCT-8 and HCT 116 cells were fractionated by gel-filtration chromatography for analysis. We found that FASN immunoreactivity was detected in eluted fractions that largely overlapped with the CSN6 protein (Fig. 2d). To map the interaction domain, CSN6 was divided into CSN6-N terminal and CSN6-C terminal. We characterized that CSN6 interacts with FASN through MPN domain in co-IP studies (Fig. 2e). The MPN domain plays an indispensable role in CSN6-mediated Cullin-1 neddylation,^31^ thereby involving in ubiquitination regulation. On the basis of these results, it is possible that CSN6, a protein known to be involved in targeted protein ubiquitination/stability, may affect the expression of FASN. Indeed, CSN6 overexpression increased the protein level of FASN, but not affected the mRNA expression level of FASN in several CRC cell lines (Fig. 2f). Conversely, CSN6 KD leads to downregulation of FASN (Fig. 2f).

We further showed that CSN6 KD accelerates FASN protein turnover (Fig. 2g), and that overexpressing CSN6 leads to reduced turnover rate of FASN (Fig. 2h). Consistently, CSN6 decreased the poly-ubiquitination level of FASN in a dose-dependent manner (Fig. 2i). Moreover, CSN6 used HA-ubiquitin (Ubi) K48 but not HA-Ubi K63 to cause reduction of FASN ubiquitination (Fig. 2j), indicating that CSN6-mediated deubiquitination of FASN is a K48 linkage, which generally targets protein for proteasome-mediated degradation. CSN6 with MPN domain deleted (CSN6-C-ter) has attenuated its activity in decreasing FASN ubiquitination, thereby losing the impact in increasing the steady-state expression of FASN (Fig. 2k, l). These data suggested that CSN6 attenuates ubiquitin-mediated degradation of FASN, thereby stabilizing FASN protein.

### CSN6 regulates FASN protein stability by antagonizing E3 ligase activity of FBXW7β toward FASN to regulate lipogenesis

**Fig. 3:**
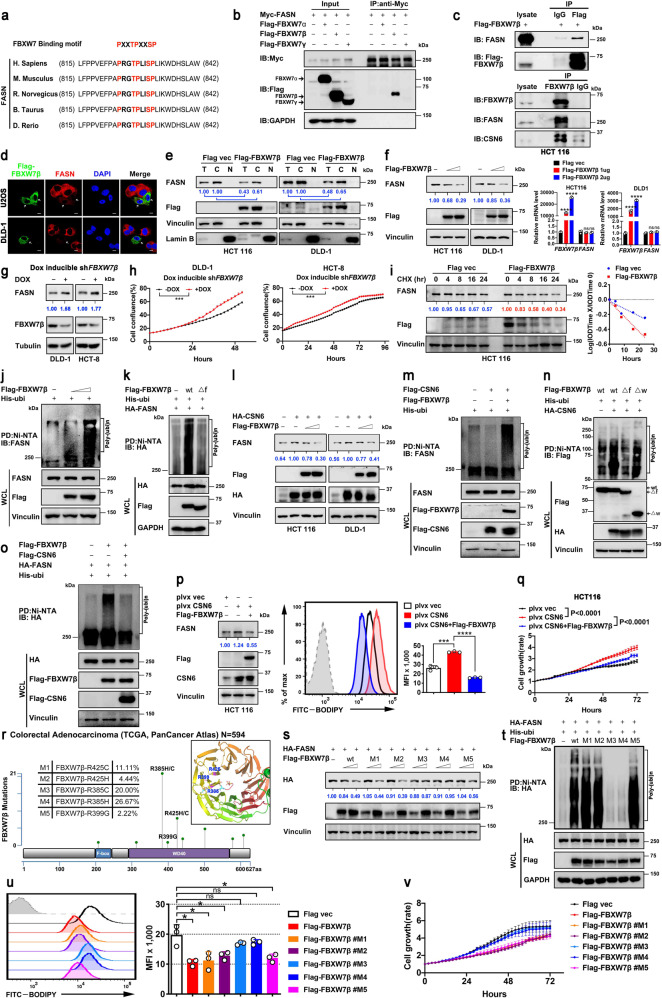
CSN6 positively regulates FASN protein stability by antagonizing the E3 ligase activity of FBXW7β toward FASN to enhance lipogenesis. **a** Peptide sequence alignment of FASN with the phospho-degron sequences recognized by FBXW7. The putative FBXW7 phospho-degron sequence present in FASN is conserved across different species. X denotes any kind of residue. **b** HEK293T cells were transfected with Myc-FASN together with the indicated Flag-tagged FBXW7. Cell lysates were immunoprecipitated with anti-myc beads and immunoblotted with the indicated antibodies. **c** Flag-FBXW7β were transfected into HEK293T cells. Cell lysates were immunoprecipitated with anti-Flag M2 agarose beads and immunoblotted with the indicated antibodies (top). HCT 116 cell lysates were immunoprecipitated with anti-FBXW7β antibodies followed by immunoblotting with the indicated antibodies (bottom). **d** Flag-FBXW7β were expressed in U2OS and DLD-1 cells by transient transfection. Immunofluorescence analyses were performed with anti-Flag-FBXW7β (green) and anti-FASN (red) antibodies. Nuclei are stained with DAPI (blue). The white arrows indicate Flag–FBXW7β-positive cells. Scale bar, 10 μm. **e** Whole cell lysate (T), the cytosolic (C) and the nucleus (N) fractions were prepared from HCT 116 or DLD-1 cells transfected with or without Flag–tagged FBXW7β. Cellular fractions from equal numbers of cells were analyzed using immunoblotting with the indicated antibodies. **f** Western blot analysis of FASN protein in cells transfected with increased dose of Flag-tagged FBXW7β (left). qRT-PCR analysis was performed to measure the mRNA expression of *FASN* (right). **g** Western blot analysis of FASN protein in indicated dox-inducible sh*FBXW7β* cells treated with or without doxycycline (DOX, 200 μg/mL). **h** Cell growth assay for indicated dox-inducible sh*FBXW7β* cells treated with or without doxycycline (DOX, 200 μg/mL). **i** HCT 116 cells transfected with or without Flag-tagged FBXW7β were treated with cycloheximide (CHX, 100 μg/mL) for the indicated time points. Cell lysates were immunoblotted with indicated antibodies (left). The relative abundance of remaining FASN protein was normalized to the *t* = 0 controls (right). **j**, **k**, **m**, **o** HEK293T cells were transfected with indicated plasmids. MG132 (20 μM) was added to the cells 6 h before they were harvested. The ubiquitinated FASN proteins were pulled down with nickel beads (Ni-NTA) and immunoblotted with an anti-FASN antibody (**j**, **m**) or anti-HA antibody (**k**, **o**). **l** HCT 116 or DLD-1 cells were transfected with the indicated plasmids. Cell lysates were immunoblotted with the indicated antibodies. **n** HEK293T cells were transfected with indicated plasmids. MG132 (20 μM) was added to the cells 6 h before they were harvested. The ubiquitinated Flag-FBXW7β proteins were pulled down with nickel beads (Ni-NTA) and immunoblotted with an anti-Flag antibody**. p** HCT 116 cells were transfected with indicated plasmids and then stained with BODIPY 493/503 for quantification of intracellular lipids. The expression level of FASN, FBXW7β and CSN6 were analyzed. Representative histograms and mean fluorescence intensities (MFI) of BODIPY were shown. **q** Cell growth assay generated from HCT 116 cells stably transfected with a control vector (plvx vec) or a vector expressing CSN6 (plvx-CSN6) and then co-transfected with or without Flag-FBXW7β. **r** Missense mutation diagram of FBXW7β in TCGA colorectal adenocarcinoma database (*N* = 594). Each circle is determined with respect to the mutation site and case number. Three mutational hot spots site located in WD40 domain of FBXW7β are shown in 3D structure. Five cancer-derived mutants of FBXW7β (M1-M5) are selected and constructed for latter experiment. **s** HEK293T cells were transfected with HA-FASN together with or without Flag-FBXW7β wild type (wt) and the indicated FBXW7β mutants (M1-M5). Cell lysates were immunoblotted with the indicated antibodies. **t** HEK293T cells were transfected with indicated plasmids. MG132 (20 μM) was added to the cells 6 h before they were harvested. The ubiquitinated FASN proteins were pulled down with nickel beads (Ni-NTA) and immunoblotted with anti-HA antibody**. u** Flow cytometry analysis of BODIPY 493/503-stained HCT 116 cells transfected with indicated plasmids. Representative histograms and mean fluorescence intensities (MFI) were shown. **v** Cell growth assay of HCT 116 cells transfected with or without Flag-FBXW7β wild type (wt) and the indicated FBXW7β mutants (M1-M5). All values are expressed as means ± SD. ns not significant, *P < 0.05, ***P < 0.001, ****P < 0.0001; as determined by two-way ANOVA (**h**, **q**, **v**) or by one-way ANOVA (**f**, **p**, **u**)

Given that CSN6 expression seems to correlated with the steady-state expression of FASN (Fig. 2), we sought to uncover how CSN6 impacts on FASN expression. To characterize a specific E3 ligase that governs FASN protein ubiquitination and degradation, we analyzed the FASN amino acid sequence and found that FASN contains an evolutionarily conserved FBXW7 binding motif (PXXTPXXSP) (Fig. 3a). FBXW7 exists in three splice-variant isoforms (α, β, γ) with specific subcellular localization. FBXW7β is cytosolic; therefore, we focused on its interaction with FASN (located in the cytoplasm). Indeed, co-IP studies demonstrated that FASN could interact with β form of FBXW7 but not the α or γ form of FBXW7 (Fig. 3b). Importantly, CSN6 is also present in FBXW7β/FASN immunoprecipitated complex (Fig. 3c). IF analysis showed that FBXW7β and FASN are located in the cytoplasm (Fig. 3d). Importantly, IF analysis of transfected U2OS or DLD-1 cells showed a striking decreased signal in cytoplasmic FASN following FBXW7β overexpression (arrowheads, Fig. 3d). Consistently, subcellular fractionation studies showed that overexpressing FBXW7β resulted in a marked decrease of FASN both in total and cytoplasmic fraction (Fig. 3e). Furthermore, ectopic expression of FBXW7β decreased the protein expression of FASN without affecting the mRNA expression of FASN (Fig. 3f). Conversely, FBXW7β KD leads to upregulation of FASN as well cell growth (Fig. 3g, h). We further showed that FBXW7β expression increased the turnover rate of FASN in a cycloheximide (CHX) assay (Fig. 3i). Importantly, FBXW7β increased the ubiquitination of FASN in a dose-dependent manner (Fig. 3j). FBXW7β is a component of SCF complex containing Skp1 and Cullin-1. F-box of FBXW7β interacts with Skp1 and is an indispensable motif for ubiquitination activity. We then showed that nonfunctional FBXW7β mutant lacking the F-box (Δf) did not increase FASN ubiquitination (Fig. 3k).

To verify the seemly counteractive impact from CSN6 and FBXW7β in regulating FASN, we performed a competition or rescue experiment. We showed that CSN6 can increase the steady-state expression (Fig. 3l) and reduce the ubiquitination of FASN (Fig. 3m), but these impacts are attenuated by the expression of FBXW7β (Fig. 3l, m). On the other hand, CSN6 increased the ubiquitination of FBXW7β (Fig. 3n); therefore, FBXW7β-mediated FASN ubiquitination level can be hindered by CSN6 overexpression (Fig. 3o). Together, these results demonstrated that CSN6 and FBXW7β participated in the vicissitude of FASN’s stability.

Next we performed a phenotypic rescue experiment, asking whether CSN6-FBXW7β relationship is involved in CSN6-mediated lipogenesis and cell growth in CRC cells. Indeed, increase lipid accumulation and tumor cell growth caused by CSN6 overexpression was observed and could be attenuated by the enforced expression of FBXW7β (Fig. 3p, q). The expression levels of each protein were confirmed by immunoblotting (Fig. 3p).

Clinically, we showed that FBXW7β gene is highly altered in colorectal adenocarcinoma based on database studies (Supplementary Fig. 2a–e). Missense mutations of FBXW7β are frequently characterized (hot spots) in colorectal adenocarcinoma (Supplementary Fig. 2a–e). Five cancer-derived hot spot mutants of FBXW7β (M1–M5 located in WD40 domain of FBXW7β, see 3D structure) were constructed for examining their impacts, especially on FASN expression (Fig. 3r). We found that M3 (R385C) and M4 (R385H) FBXW7β mutants, which are most highly mutated in several data sets (Supplementary Fig. 2a–e), have lost their activity in attenuating steady-state expression of FASN, increasing the ubiquitination of FASN, reducing BODIPY staining, and inhibiting cell growth when compared with wild type (wt) FBXW7β (Fig. 3s–v), suggesting that FBXW7β mutations in several hot spots leads to deregulation of lipogenesis in colon cancer.

### GSK3β-mediated FASN phosphorylation instigates FBXW7β-mediated FASN ubiquitination/degradation and inhibits lipogenesis

**Fig. 4:**
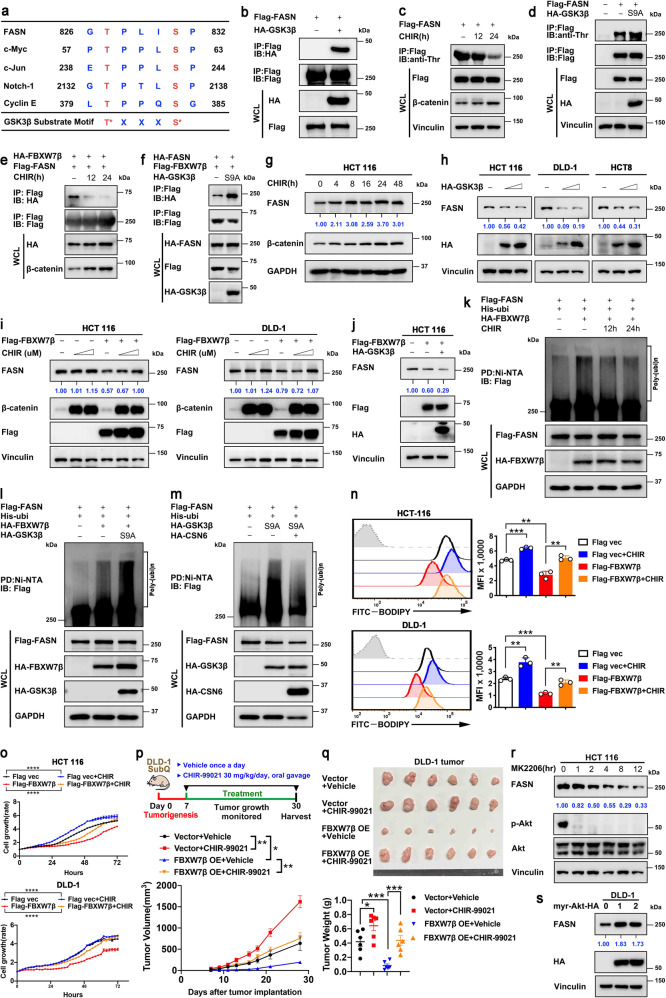
GSK3β-mediated FASN phosphorylation enhances FBXW7β-mediated ubiquitination/degradation of FASN and inhibits lipogenesis. **a** Sequence alignment of GSK3β substrate consensus sequences (S/TXXXS/T) and FBXW7β binding motifs of FASN. The reported GSK3β substrates (c-Myc, c-Jun, Notch-1, Cyclin E) are shown. Red, phospho-acceptor residue; blue, basic residue. X denotes any kind of residue. **b** HEK293T cells expressing Flag-FASN were co-transfected with HA-GSK3β. Cell lysates were immunoprecipitated with anti-Flag M2 agarose beads and immunoblotted with the indicated antibodies. **c** HEK293T cells expressing Flag-FASN were treated with GSK3β inhibitor CHIR (4 μM) for the indicated time. DMSO was used as a vehicle control. Cell lysates were immunoprecipitated with anti-Flag M2 agarose beads and immunoblotted with anti-phosphorylated-threonine (p-Thr) antibody. CHIR treatment efficiency was tested by β-catenin. **d** HEK293T cells were transfected with Flag-FASN together with or without HA-tagged constitutively active mutant (S9A) of GSK3β. Cell lysates were immunoprecipitated with anti-Flag M2 agarose beads and immunoblotted with anti-phosphorylated-threonine (p-Thr) antibody. **e** HEK293T cells were co-transfected with Flag-FASN and HA-FBXW7β. Where indicated, CHIR (4 μM) was added for 12 h or 24 h before harvesting. DMSO was used as a vehicle control. Cell lysates were immunoprecipitated with anti-Flag M2 agarose beads and immunoblotted with the indicated antibodies. **f** HEK293T cells were co-transfected with HA-FASN and Flag-FBXW7β in the presence or absence of HA-GSK3β (S9A). Cell lysates were immunoprecipitated with anti-Flag M2 agarose beads and immunoblotted with the indicated antibodies. **g** HCT 116 cells were treated with CHIR (4 μM) for the indicated time. DMSO was used as a vehicle control. Cell lysates were immunoblotted with the indicated antibodies. **h** Indicated cells were transfected with increased dose of HA-tagged GSK3β. Cell lysates were immunoblotted with the indicated antibodies. **i** HCT 116 or DLD-1 cells were transfected with or without Flag-FBXW7β. Increasing dose of CHIR (0/4/8 μM) was added for 24 h before harvest. DMSO was used as a vehicle control. Cell lysates were immunoblotted with the indicated antibodies. **j** HCT 116 were transfected with the indicated plasmids. Cell lysates were immunoblotted with the indicated antibodies. **k** HEK293T cells were transfected with the indicated plasmids and treated with CHIR (4 μM) for the indicated time. MG132 (20 μM) was added to the cells 6 h before they were harvested. The ubiquitinated FASN proteins were pulled down with nickel beads (Ni-NTA) and immunoblotted with an anti-Flag antibody. **l**, **m** HEK293T cells were transfected with the indicated plasmids. MG132 (20 μM) was added to the cells 6 h before they were harvested. The ubiquitinated FASN proteins were pulled down with nickel beads (Ni-NTA) and immunoblotted with an anti-Flag antibody. **n** HCT 116 cells transfected with indicated plasmids were treated with or without CHIR (4 μM) for 24 h and then stained with BODIPY 493/503 for quantification of intracellular lipids. Representative histograms and mean fluorescence intensities (MFI) were shown. **o** Cell growth assay generated from HCT 116 cells transfected with indicated plasmids and then treated with or without CHIR (4 μM). **p** Schematic design for DLD-1 subcutaneous tumor model (top). Tumor growth curves of indicated DLD-1 tumors (*n* = 6 mice per group) were shown (bottom). **q** The resulting tumors generated in **p** were resected and photographed at the end of the experiment (top). Tumor weights of indicated DLD-1 tumors were shown (bottom). **r** HCT 116 cells were treated with AKT inhibitor MK2206 (2 μM) for the indicated time. Cell lysates were immunoblotted with the indicated antibodies. **s** DLD-1 cells were transfected with increased dose of constitutively active form of Akt (myr-Akt). Cell lysates were immunoblotted with the indicated antibodies. All values are expressed as means ± SD. ns not significant, *P < 0.05, **P < 0.01, ***P < 0.001, ****P < 0.0001; as determined by two-way ANOVA (**o**, **p**) or by one-way ANOVA (**n**, **q**)

**Fig. 5:**
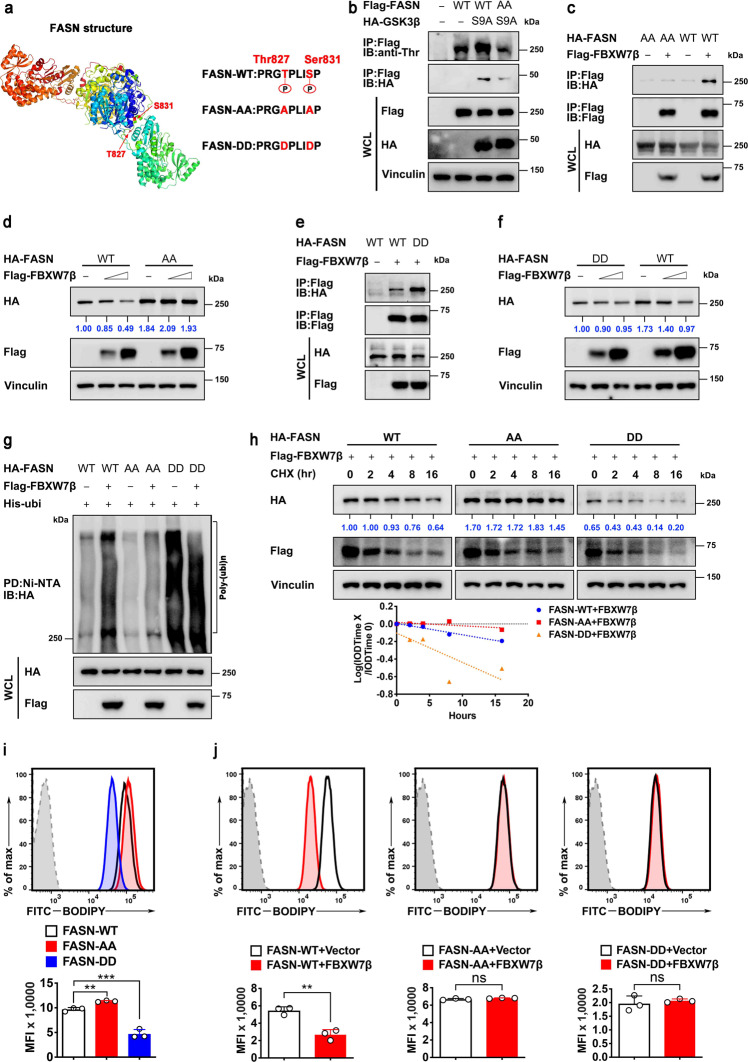
Phosphorylation of Thr827/Ser831 sites of FASN is critical for FBXW7β-mediated FASN degradation. **a** Sequences of FASN phospho-degron mutant constructs. **b** HEK293T cells were transfected with indicated Flag-FASN constructs together with or without HA-tagged GSK3β (S9A). Cell lysates were immunoprecipitated with anti-Flag M2 agarose beads and immunoblotted with anti-phosphorylated-threonine (p-Thr) antibody. **c**, **e** HEK293T cells were transfected with indicated HA-FASN constructs together with or without Flag-FBXW7β. Cell lysates were immunoprecipitated with anti-Flag M2 agarose beads and immunoblotted with the indicated antibodies. **d**, **f** HEK293T cells were transfected with the indicated HA-FASN constructs together with increasing amounts of Flag-FBXW7β. Cell lysates were immunoblotted with the indicated antibodies. Expression of HA-FASN constructs were quantitated. **g** HEK293T cells were transfected with indicated HA-FASN constructs and His-Ubi plasmids together with or without Flag-FBXW7β. The ubiquitinated FASN proteins were pulled down with nickel beads (Ni-NTA) and immunoblotted with an anti-HA antibody. **h** HEK293T cells were transfected with the indicated HA-FASN constructs together with Flag-FBXW7β. Cells were treated with cycloheximide (CHX, 100 μg/mL) for the indicated time. Cell lysates were immunoblotted with indicated antibodies (top). The relative abundance of remaining FASN protein was normalized to the *t* = 0 controls (bottom). **i**, **j** HEK293T cells were transfected with the indicated plasmids and then stained with BODIPY 493/503 for quantification of intracellular lipids. Representative histograms and mean fluorescence intensities (MFI) were shown. The data are presented as the means ± SD as determined by one-way ANOVA (**i**) or by two-sided Student’s *t*-test (**j**). ns not significant, **P < 0.01, ***P < 0.001

FBXW7 targets typically contain the FBXW7 binding motif (degron) that includes phosphorylated serine or threonine residues in the “0” and “+4” positions. GSK3β phosphorylates this motif to facilitate FBXW7 recognition (Fig. 4a). Analysis of FASN sequence indeed revealed a FBXW7β-binding motif containing well-characterized GSK3β phosphorylation sites (827TXXXS831) (Figs. 4a, 5a). Co-IP studies indicated the interaction between GSK3β and FASN (Fig. 4b). Phosphorylation studies indicated that FASN can be Thr-phosphorylated, whereas treatment with a GSK3β inhibitor CHIR markedly reduced FASN phosphorylation level (Fig. 4c). Consistently, constitutively active form of GSK3β (S9A) overexpression increased Thr-phosphorylation of FASN (Fig. 4d).

GSK3β inhibitor (CHIR) dramatically reduced the association between FASN and FBXW7β (Fig. 4e), while GSK3β (S9A) strengthens the association (Fig. 4f). Time-course studies revealed that treatment with the GSK3β inhibitor CHIR significantly increased the protein level of FASN (Fig. 4g). On the other hand, GSK3β expression reduced the steady-state expression of FASN in a dose-dependent manner (Fig. 4h). To put FBXW7β in the context, we found that FBXW7β-mediated FASN degradation can be antagonized by GSK3β inhibitor CHIR (Fig. 4i), while FBXW7β-mediated FASN downregulation is strengthened by the expression of GSK3β (Fig. 4j). Congruently, FBXW7β-mediated FASN ubiquitination is attenuated by GSK3β inhibitor CHIR (Fig. 4k), while FBXW7β-mediated FASN ubiquitination is boosted by the expression of GSK3β (S9A) (Fig. 4l). Finally, to link CSN6 to the event, we showed that GSK3β (S9A)-enhanced FASN ubiquitination is attenuated by the expression of CSN6 (Fig. 4m), suggesting that CSN6 antagonized FBXW7β/GSK3β-mediated FASN ubiquitination.

To determine the phenotypic effect of GSK3β in governing FBXW7β-mediated FASN degradation. We detect cellular lipid synthesis and cell growth driven by FBXW7β-GSK3β axis in CRC cell lines. Interestingly, treatment with GSK3β inhibitor CHIR can rescue the lipid accumulation defect (Fig. 4n) or reverse cell growth/tumor inhibition caused by the expression of FBXW7β (Fig. 4o–q). Since GSK3β is a downstream target of AKT and is inhibited by AKT activation, we expected that Akt signaling blockage would improve GSK3β activity, resulting in increased FASN phosphorylation and degradation. In line with this possibility, AKT inhibitor MK2206 reduced the steady-state expression of FASN in a time-dependent manner (Fig. 4r), while constitutively active form of Akt (myr-Akt) expression increased the steady-state expression of FASN (Fig. 4s).

To further investigate the crucial role of the phospho-degron of FASN in FBXW7β-mediated degradation of FASN, we mutated FASN phosphorylation site by displacing Thr827 and Ser831 sites with alanine (FASN-AA) or aspartic acid (FASN-DD) (Fig. 5a). GSK3β-mediated phosphorylation on FASN-AA is compromised when compared with FASN-WT (Fig. 5b). Thus, FASN-AA mutant failed to bind FBXW7β (Fig. 5c), indicating that the phosphorylation on Thr827/ Ser831 sites is crucial for FASN recognition by FBXW7β. Because FASN-AA mutant fails to bind FBXW7β, FASN-AA mutant is resistant to FBXW7β-mediated downregulation (Fig. 5d). On the other hand, FASN-DD mutant enhanced its interaction with FBXW7β when compared to FASN-WT (Fig. 5e). Steady-state expression of FASN-DD mutant is quickly decreased when compared to FASN-WT as expected (Fig. 5f). These characteristics of FASN-AA mutant were translated into its resistance to FBXW7β-mediated poly-ubiquitination (Fig. 5g) and its reduced turnover rate (Fig. 5h) when compared with WT FASN control. Conversely, phosphorylation mimetic FASN-DD mutant has enhanced FBXW7β-mediated poly-ubiquitination (Fig. 5g) and accelerated turnover rate (Fig. 5h) when compared with WT FASN control.

Congruently, FASN-AA mutant increased cellular lipid accumulation when compared with FASN-WT based on BODIPY staining by flow cytometry, while FASN-DD mutant has lower lipid amount when compared with FASN-WT (Fig. 5i). Further, FASN-WT-mediated cellular lipid accumulation can be attenuated by the overexpression of FBXW7β based on BODIPY staining intensity, but FASN-AA mutants are resistant to FBXW7β’s impact (Fig. 5j). The FASN-DD mutants already have small amounts of cellular lipid accumulation based on BODIPY staining intensity scale when compared with FASN-WT, and are not further affected by FBXW7β’s impact (Fig. 5j, right). Together, these data establish the regulation of FBXW7β-FASN phosphorylation/degradation-cellular lipid synthesis axis.

### CSN6 enhances FBXW7β autoubiquitination and degradation

To understand the detailed role of CSN6 and FBXW7β in regulating FASN stability, we investigate the regulation between CSN6 and FBXW7β. CSN6 is known to associate with several E3 ligase. The co-IP experiment showed the endogenous interaction between CSN6 and FBXW7β (Fig. 3c). Congruently, we further confirmed their exogenous interaction in a co-IP study (Supplementary Fig. 3a). The significance of this interaction was demonstrated in that dox-induced CSN6 KD led to increased steady-state-expression of FBXW7β with concurrent decreased expression of FASN (Supplementary Fig. 3b), while CSN6 overexpression reduced FBXW7β and resulted in increased FASN level (Supplementary Fig. 3c). We conducted transcription and translation (TNT) assay to produce the proteins (FBXW7β, CSN6 and FASN); in vitro co-IP studies showed that FBXW7β and CSN6 have direct binding and that FBXW7β and FASN have direct binding. However, CSN6 and FASN have no direct binding (Supplementary Fig. 3d). We examined gel-filtration chromatography fractions from dox inducible sh*CSN6*#1 HCT 116 and found that CSN6 KD cell extracts had more FBXW7β expression with concurrent decreased FASN expression than control cell extracts when compared fraction by fraction (Supplementary Fig. 3e). Further, CSN6 KD reduced the turnover rate of FBXW7β (Supplementary Fig. 3f), and overexpressing CSN6 increased the ubiquitination of FBXW7β (Supplementary Fig. 3g). These data suggest that CSN6 decreases FBXW7β protein level through ubiquitination regulation in order to prevent FBXW7β-mediated FASN degradation.

FBXW7, including FBXW7β, is rapidly degraded by an autoubiquitination mechanism. We then hypothesized that CSN6 enhances this autoubiquitination process of FBXW7β. Domain mapping studies indicated that CSN6 associates with FBXW7β through MPN domain containing N-Ter (Supplementary Fig. 3h, i). Thus, we showed that CSN6 with MPN domain deleted (CSN6-C-ter) has lost its activity in mediating FBXW7β ubiquitination (Supplementary Fig. 3j). Further, we constructed several FBXW7β domain mutants (Supplementary Fig. 3k) and showed that F-box domain of FBXW7β is required to interact with Cullin-1 (Supplementary Fig. 3l). It has been noted that F-box of FBXW7β is critical for its interaction with Cullin-1 and subsequent autoubiquitination. Interestingly, CSN6 can only increase the poly-ubiquitination of wt FBXW7β and FBXW7βΔW (WD40 domain deleted) but not FBXW7βΔf (F-box deleted) (Fig. 3n). These data suggest that CSN6 increases FBXW7β ubiquitination through enhancing autoubiquitination of FBXW7β, thereby reducing the steady-state expression of FBXW7β. These data demonstrate that CSN6-mediated FBXW7β autoubiquitination and degradation involve MPN domain of CSN6 and F-box domain of FBXW7β.

### CSN6-induced lipogenesis facilitates CRC tumor growth, which can be suppressed by FASN inhibitor orlistat

**Fig. 6:**
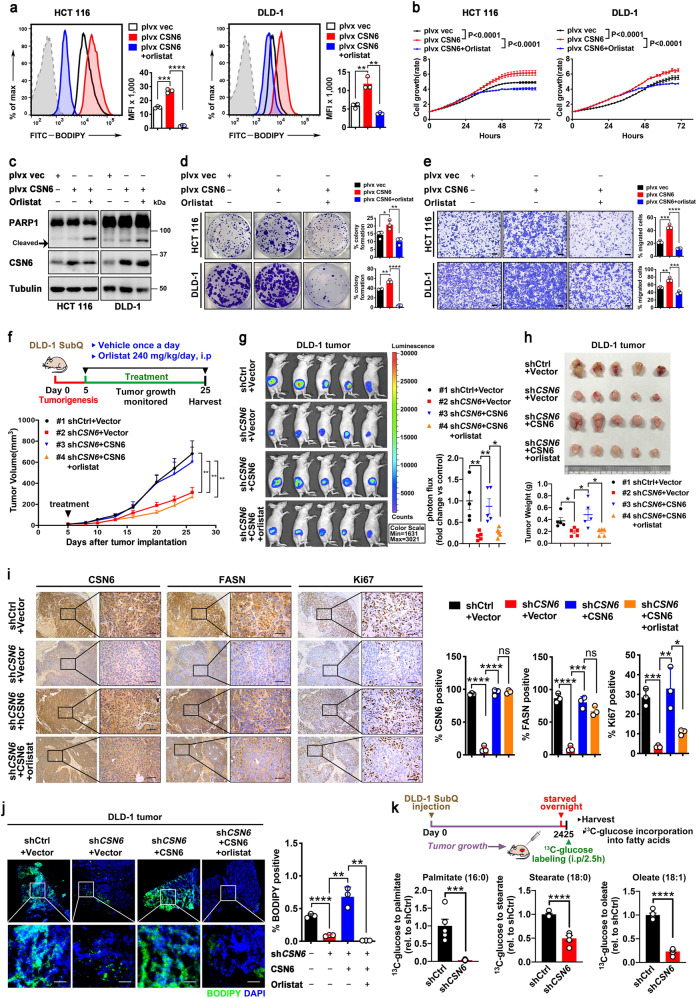
CSN6/FASN-induced lipogenesis promotes CRC tumor growth, which can be inhibited by orlistat. **a** The effect of orlistat on CSN6-mediated lipogenesis was determined by BODIPY 493/503 staining followed by flow cytometry analysis. Representative histograms and mean fluorescence intensities (MFI) in HCT 116 (left) and DLD-1 (right) cells were shown. **b** Cell growth assays for HCT 116 (left) and DLD-1 (right) cells stably transfected with indicated plasmids and then treated with vehicle (DMSO) or orlistat (8 μM). **c** Western blot analysis for cleaved PARP1 expression in HCT 116 and DLD-1 cells stably transfected with indicated plasmids and then treated with vehicle (DMSO) or orlistat (8 μM). Arrow denotes molecular weight of cleaved PARP1. **d**, **e** Colony-formation assays (**d**) and migration assays (**e**) generated from HCT 116 and DLD-1 cells stably transfected with indicated plasmids and then treated with vehicle (DMSO) or orlistat (8 μM). Scale bar, 200 μm. **f** Scheme of experimental design for DLD-1 subcutaneous tumor model (top). Tumor growth curves of indicated DLD-1 tumors (*n* = 5 mice per group) were shown (bottom). **g** Bioluminescence overlay images of indicated DLD-1 tumors in mice after 28 days growth (left). Colored scale shows photon flux in units of radiance (photons/s/cm^2^/steradian(sr)). Quantification of photon flux for the indicated DLD-1 tumors was shown (right). **h** The resulting tumors generated in **f** were resected and photographed at the end of the experiment (top). Tumor weights of indicated DLD-1 tumors were shown (bottom). **i** Representative IHC images of CSN6, FASN and Ki67 staining in indicated DLD-1 tumors (left). The regions in black boxes are shown at higher magnification. Scale bars, 50 μm. Quantification of the percentage of CSN6 positive, FASN positive and Ki67 positive cells of indicated groups was shown (right). **j** Fluorescence microscopy analysis of frozen sections from indicated DLD-1 tumors stained with BODIPY 493/503 dye (green) and DAPI (blue). Representative images (left) and fluorescent BODIPY quantification (right) of indicated groups were shown. Scale bar, 20 μm. **k** Schematic design for in vivo fatty acid metabolite tracing of indicated DLD-1 tumors (top). Ratio of ^13^C-Glucose to indicated fatty acid metabolites was shown (bottom). All values are expressed as means ± SD. ns not significant, *P < 0.05, **P < 0.01, ***P < 0.001, ****P < 0.0001; as determined by two-way ANOVA (**b**, **f**), by one-way ANOVA (**a**, **d**, **e**, **g**–**j**) or by two-sided Student’s *t*-test (**k**)

Previous studies have shown that CSN6 is involved in the process of promoting colorectal malignancy.^8^ To determine the role of FASN in CSN6-promoted proliferation of CRC cells, we analyzed cellular growth of CSN6-expressing cells in the presence of FASN inhibitor orlistat (an FDA-approved anti-obesity drug). CSN6-mediated lipogenesis and cell proliferation effect were attenuated by orlistat treatment (Fig. 6a, b). Also, CSN6-expressing cells treated with orlistat can lead to increased apoptosis as demonstrated by apoptosis marker cleaved PARP1 and Annexin IV/PI staining (Fig. 6c and Supplementary Fig. 4a). CSN6-mediated enhancing cancer cell colony formation and migration can also be reversed by orlistat (Fig. 6d, e). In addition to orlistat, we evaluated another FASN inhibitor C75 for its effect on tumor growth advantage driven by CSN6. Similar to the observations made after orlistat treatment, C75 treatment group demonstrated a robust inhibition in cell proliferation, induced PARP1 cleavage, attenuated colony formation and migration capacity in CSN6-expressing cells (Supplementary Fig. 4b–e).

To determine the in vivo contribution of CSN6-FASN-lipogenesis axis in promoting tumor growth, we performed DLD-1 cell growth and mouse xenograft cancer studies. CSN6 KD leads to inhibition of cell growth and tumor volume, but additional CSN6 (shRNA resistant)-reexpression rescued this impact caused by CSN6 KD as demonstrated by promoting cell growth and increasing tumor volume (Fig. 6f, Supplementary Fig. 4f), enhancing tumor bioluminescent intensity (Fig. 6g), and increasing final tumor weight (Fig. 6h). Again, this rescued impact of reexpression of CSN6 in promoting the tumor growth can be reversed by orlistat treatment (Fig. 6f–h). Orlistat treatment did not significantly alter the mouse body weight during the course of the experiment (Supplementary Fig. 4g).

Further, CSN6 KD tumors demonstrate decreased FASN protein levels based on immunohistochemistry (IHC) staining (Fig. 6i) and immunoblotting (Supplementary Fig. 4h), low BODIPY staining (Fig. 6j) and relatively low levels of tumor cell growth (as measured by Ki67 staining) (Fig. 6i), while CSN6-reexpressing tumors can restore the levels of FASN (Fig. 6i, Supplementary Fig. 4h), increase BODIPY staining (Fig. 6j), and elevate Ki67 staining concurrently (Fig. 6i). The administration of orlistat in rescue group attenuated the elevation of Ki67 staining and BODIPY staining (Fig. 6i, j).

In a separate in vivo metabolite tracing experiment using ^13^C-glucose also demonstrates that CSN6 KD tumors contained diminished levels of palmitate (16:0), stearate (18:0) and oleate (18:1) based on metabolite tracing when compared with controlled tumors (Fig. 6k). Together, the correlation between CSN6, FASN, and de novo lipid synthesis could be recapitulated in mouse xenograft cancer study, and tumor suppressive impact of orlistat in inhibiting FASN and lipid synthesis can be explored in hindering CSN6-overexpressing tumorigenicity.

### High levels of FASN and CSN6 correlate with poor survival of CRC, suggesting a treatment strategy of orlistat administration

To further validate CSN6-FASN axis in terms of clinical significance, we analyzed the protein levels of FASN and CSN6 in CRC cell lines or tumor tissues. First, analysis of protein abundance of CSN6 and FASN in one normal colon cell line (HCEC) and ten selected FBXW7β-wild type (wt) CRC cell lines indicated that CSN6 and FASN levels were elevated in various CRC cell lines and that their expression was positively correlated (Supplementary Fig. 5a–c, Supplementary Table 2). An analysis of the protein expression data from the CRC set in The Cancer Proteome Atlas (TCPA) and Clinical Proteomic Tumor Analysis Consortium (CPTAC) databases also indicated that FASN was overexpressed in cancer tissue when compared with normal tissue (Supplementary Fig. 5d); again the FASN levels were positively correlated with the levels of CSN6 (Supplementary Fig. 5e).

We collected paired adjacent normal (N) and tumor (T) tissue samples from 12 CRC patients, and the results showed that the expression of both CSN6 and FASN was elevated in tumor tissues compared to adjacent normal tissues (Supplementary Fig. 5f) and that their expression level was positively correlated in tumor tissues (Supplementary Fig. 5f). We also employed tissue microarray (TMA) samples for further studies. A testing cohort including paired samples of normal colon and CRC from 72 patients was analyzed. Expression of both CSN6 and FASN in CRC was higher than that in adjacent normal tissue based on IHC staining (Supplementary Fig. 5g).

**Fig. 7:**
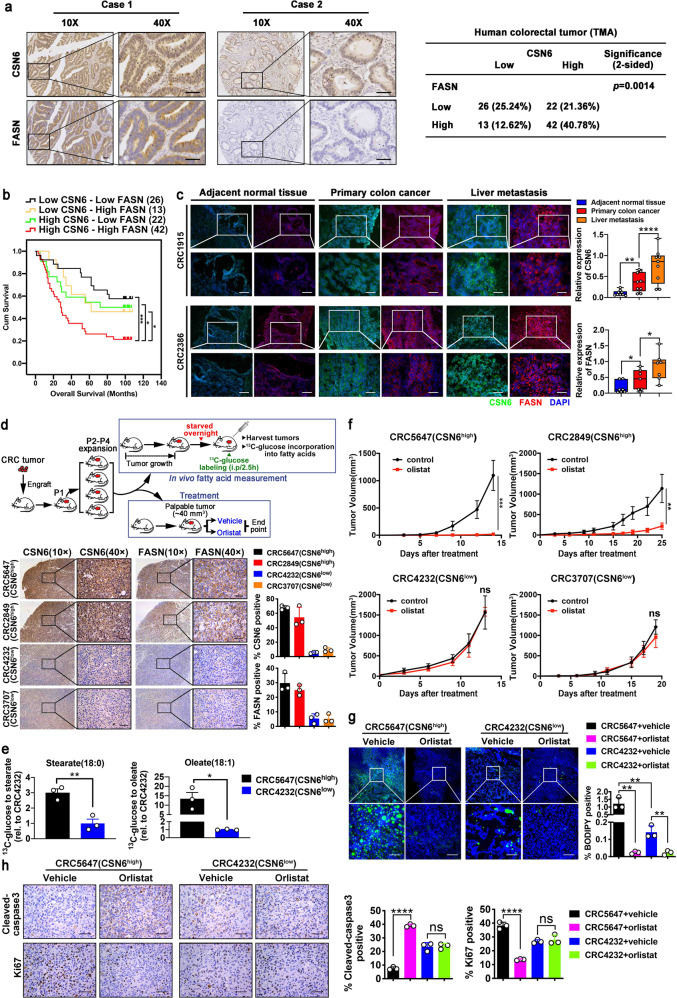
High levels of CSN6/FASN correlate with poor survival of CRC, suggesting a treatment strategy of orlistat administration. **a** Representative IHC images of CSN6 and FASN in human 103 CRC tissue microarray samples (left). Scale bars, 50 μm. Correlation of FASN and CSN6 staining through statistical analysis was shown (right). **b** Kaplan–Meier survival curves of overall survival time based on CSN6 and FASN expression from TMA analysis. The receiver operating characteristic curve was used to define the cutoff, and log-rank analysis was used to test for significance. **c** Representative immunofluorescent images for CSN6 (green) DAPI (Blue), and FASN (red) in frozen section of colorectal cancer tissue of primary site, adjacent normal tissue, and liver metastasis tissue from same colorectal cancer patient (left). Relative protein expression was quantitated by Image J and presented as bar graphs (right). Scale bar, 50 μm. **d** Scheme of experimental design for colorectal patient-derived xenografts (PDXs), treatment schedule of FASN inhibitor orlistat and in vivo fatty acid measurement of tumors is indicated (top). P1, passage 1; P2, passage 2; P3, passage 3. Representative IHC images of CSN6 and FASN in indicated PDXs (bottom). Quantification of CSN6 and FASN expression was indicated. **e** Relative levels of Stearate/Oleate based on ^13^C-glucose metabolite tracing in two PDX cases. **f** Impact of orlistat on tumor growth in mice bearing indicated PDXs. **g** Fluorescence microscopy analysis of frozen sections from indicated PDX tumors stained with BODIPY 493/503 dye (green) and DAPI (blue). Representative images (left) and fluorescent BODIPY quantification (right) of indicated groups were shown. Scale bar, 20 μm. **h** Representative IHC images of cleaved-caspase 3 and Ki67 staining in indicated PDX tumors (left). Scale bars, 50 μm. Quantification of the percentage of Cleaved-caspase 3 and Ki67 positive cells of indicated groups was shown (right). All values are expressed as means ± SD. ns, not significant, *P < 0.05, **P < 0.01, ***P < 0.001, ****P < 0.0001; as determined by two-way ANOVA (**f**), by one-way ANOVA (**c**, **d**, **g**, **h**) or by two-sided Student’s *t*-test (**e**)

Significantly, we found a strong positive correlation between CSN6 and FASN expression as analyzed in 103 CRC tissue microarrays in another cohort by IHC analysis (Fig. 7a). Quantitation analysis showed that patient with strong CSN6 IHC staining also had a strong FASN staining (Fig. 7a). Kaplan–Meier survival analysis revealed that CRC patients with high expression of both CSN6 and FASN had a poor overall survival when compared with those with low expression of CSN6 and FASN (Fig. 7b). Concurrently, we analyzed the clinical characteristics of CRC patients, and the results showed that high CSN6/FASN expression was positively correlated with advanced histological grade of CRC patients (Supplementary Table 3). Moreover, univariate and multivariate Cox regression analysis were performed (Supplementary Table 4). These data demonstrate that CSN6 expression was positively correlated with FASN in colorectal cancer and responsible for poor prognoses.

Liver metastasis is often observed in CRC and leads to the high mortality and morbidity rate in CRC patients. Importantly, metastatic liver cancer samples from CRC patients demonstrated higher levels of FASN and CSN6 when compared with colorectal cancer of primary site and its adjacent normal tissue (Fig. 7c), indicating the role of CSN6/FASN in promoting metastasis.

To further explore whether controlling CSN6-FASN axis can mitigate the tumor development in CRC patients, we established patient-derived xenografts (PDXs)^44,45^ by transplanting fresh primary CRC specimens into the NCG mice to analyze tumor growth (Fig. 7d). CSN6 KD (or low level) seems to be insensitive to the growth inhibitory effect of orlistat (Supplementary Fig. 6a), which serves as a rationale for exploring CSN6 status in orlistat-mediated tumor suppression. Several sets of CRC PDX were established, and CSN6/FASN expression levels were assessed (Supplementary Fig. 6b). Two PDX cases containing high CSN6 with concurrent high FASN (CRC5647, CRC2849) and two other PDX cases containing low CSN6 with concurrent low FASN (CRC4232, CRC3707) were selected for tumor growth studies under the orlistat treatment (Fig. 7d). Representative CSN6-high PDX was shown to contain high stearate and oleate (Fig. 7e), suggesting impact from high CSN6-mediated FASN expression.

Significantly, administration of orlistat in the high CSN6/FASN PDXs can hinder tumor growth as demonstrated by reducing tumor volume (Fig. 7f). Conversely, orlistat had a minimal effect on the tumor growth of CSN6-low PDXs (Fig. 7f).

The administration of orlistat in representative high CSN6/FASN PDX dramatically inhibited the lipid droplets formation (BODIPY 493/503 staining) (Fig. 7g), increased caspase 3 cleavage (Fig. 7h), decreased Ki67 staining (Fig. 7h), and reduced tumor weight (Supplementary Fig. 6c), while orlistat’s impact on representative CSN6 low group is less impressive (Fig. 7g, h, Supplementary Fig. 6c). These data indicate that orlistat treatment can be a therapeutic strategy for high CSN6/FASN CRC patients.

### Combination treatment of cetuximab and orlistat suppressed CSN6-high PDX tumor growth with better efficacy than cetuximab or orlistat alone

**Fig. 8:**
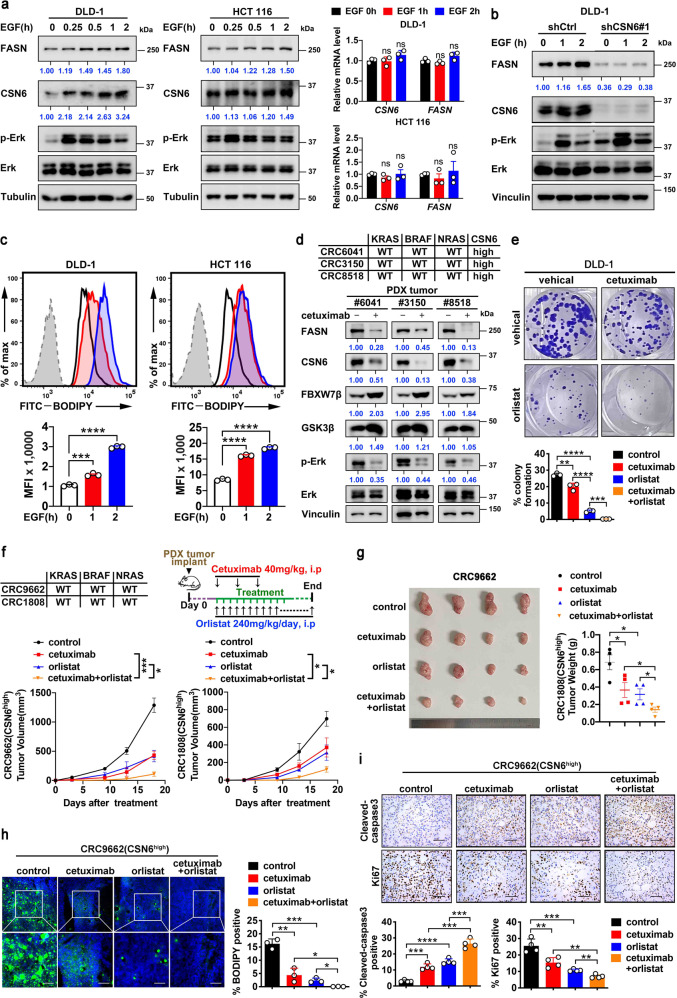
Combination treatment of cetuximab and orlistat suppressed CSN6-high CRC PDX model tumor growth with better efficacy. **a** DLD-1 and HCT 116 cells were serum-starved overnight and treated with EGF (100 ng/mL) for the indicated time. Cell lysates were immunoblotted with the indicated antibodies (left). qRT-PCR analysis was performed to measure the mRNA expression of indicated genes (right). **b** DLD-1 cells infected with control shRNA or CSN6 shRNA were treated with EGF (100 ng/mL) for the indicated time. Cell lysates were immunoblotted with the indicated antibodies. **c** Flow cytometry analysis of BODIPY-stained DLD-1 and HCT 116 cells treated with EGF (100 ng/mL) for the indicated time. Representative histograms and mean fluorescence intensities (MFI) were shown. **d** Gene status of CRC PDXs is indicated (top). Western blot analysis of indicated proteins in PDX tumors with or without cetuximab treatment (bottom). **e** Colony-formation assays generated from DLD-1 cells treated with cetuximab and/or orlistat are indicated. **f** Gene status of CRC PDXs and treatment schedule of cetuximab and/or orlistat are indicated (top). The growth curve of indicated PDX tumors in mice was measured (bottom). **g** The resulting tumors generated in **f** were resected and photographed at the end of the experiment (top). Tumor weight of each group was measured (bottom). **h** Fluorescence microscopy analysis of frozen sections from indicated PDX tumors stained with BODIPY 493/503 dye (green) and DAPI (blue). Representative images (left) and fluorescent BODIPY quantification (right) of indicated groups were shown. Scale bar, 20 μm. **i** Representative IHC images of cleaved-caspase 3 and Ki67 staining in indicated PDX tumors (top). Scale bars, 50 μm. Quantification of the percentage of Cleaved-caspase 3 and Ki67 positive cells of indicated groups was shown (bottom). All values are expressed as means ± SD. ns, not significant, *P < 0.05, **P < 0.01, ***P < 0.001, ****P < 0.0001; as determined by two-way ANOVA (**f**) or by one-way ANOVA (**a**, **c**, **e**, **g**–**i**)

Our previous studies have shown that EGF activity is frequently elevated in CRC, and that EGF regulates CSN6 expression posttranscriptionally.^8^ We hypothesized that EGF signal might play a role in modulating CSN6/FASN expression. Indeed, we found that EGF treatment in DLD-1 and HCT 116 cells can activate ERK and increase the expression of both FASN and CSN6 without affecting the mRNA expression (Fig. 8a). EGF-mediated upregulation of FASN can be compromised by the CSN6 KD, suggesting the important role of CSN6 in this process (Fig. 8b). Moreover, we performed BODIPY staining on these cells, and found that EGF treatment led to increased lipid accumulation (Fig. 8c). Because of the involvement of EGF, we then examined the impact of anti-EGFR monoclonal antibody cetuximab on the expression of CSN6 and FASN and found that cetuximab reduced the expression of both CSN6 and FASN with concurrent p-Erk inhibition and FBXW7β elevation in several cases of PDX (Fig. 8d). EGFR inhibitor cetuximab is commonly employed in CRC treatment. However, only half of them respond well, which suggested potential uncharacterized activated oncogenic signals that are responsible for 50% of ineffectiveness. We then propose to use the combination of orlistat and cetuximab for cancer cell growth inhibition. Indeed, combination treatment of cetuximab and orlistat had a better effect in hindering cell growth than orlistat or cetuximab alone, as determined by colony formation assay in DLD-1 cells (Fig. 8e). Next, we established a CRC PDX model for drug efficacy test (Fig. 8f). Significantly, administration of the FASN inhibitor orlistat or cetuximab alone impeded the tumor volume and tumor mass of CSN6-high PDXs with some efficacy, while a combination of these two drugs was more efficient in suppressing tumor growth (Fig. 8f, g). Moreover, drug combination led to a significant decreased lipid accumulation (Fig. 8h), elevated caspase 3 cleavage (Fig. 8i), and reduced Ki67 staining (Fig. 8i). The efficacy of drug treatment was verified by western blotting (Supplementary Fig. 6d).

Together, our studies demonstrate that the role of EGF/CSN6 in increasing FASN accumulation and promoting CRC malignancy can be recapitulated in clinical samples. Furthermore, we demonstrated the regulatory circuit among EGF signaling, CSN6 signaling, FASN expression level, and accumulation of fatty acids during cancer formation. Our PDX experiments prove the effectiveness of employing orlistat and cetuximab combination in hindering tumor growth for CSN6-high CRC.

## Discussion

CSN6 participates in regulating cell cycle control, signal transduction, and tumorigenesis,^46^ but its role in lipid metabolism has not been documented. Cancers have frequent lipid metabolism deregulation, but the underlying mechanism remains largely unknown.^47^ Here we characterize that CSN6 is an important regulator of lipogenesis involved in cell antagonizing apoptosis and promoting growth of CRC. We showed that EGF, CSN6, GSK3β and FBXW7β participate in regulating FASN protein expression. FBXW7β, a tumor suppressor and E3 ligase component, is frequently deregulated in CRC, and its targets are poorly defined. Here we characterize that E3 ligase coordinator CSN6, GSK3β kinase, and cytosolic FBXW7β have activities in modulating FASN protein stability through ubiquitination and proteasome-mediated degradation. FBXW7β is characterized as a specific E3 ligase that recognizes and degrades FASN upon phosphorylation of FASN by the Akt-GSK3β axis. Notably, CSN6 negatively regulates FBXW7β through enhancing FBXW7β autoubiquitination, thereby stabilizing FASN. Our data reveal upstream regulation layers of FASN and illustrates how oncogenic signals from EGF-CSN6 link FBXW7β-FASN axis for promoting lipogenesis and tumorigenesis in cancer (Supplementary Fig. 7).

FASN regulation will affect its upstream metabolites and downstream metabolites, which in turn impacts on several important biological functions involved in cancer or disease. For examples, FASN inhibition leads to mTOR lysine malonylation through metabolite signaling,^5^ which in turn causes impaired mTORC1 kinase activity and reduces phosphorylation of downstream targets. Further, fatty acid synthase can mediate EGFR palmitoylation, resulting in tyrosine kinase inhibitor (TKI) resistance in lung cancer.^48^ Our studies showed that CSN6-FASN axis increase palmitate production, implying that CSN6 may increase EGFR palmitoylation and possibly causes TKI-drug resistance. Also, FASN is critical in the maintenance of Glioma stem cell stemness^49^ and is involved in tumor growth and invasion in glioblastoma. Together, it is then critical to understand the role of FASN deregulation in cancer to find a better strategy for cancer treatment. c-Myc can regulate FASN through transcriptional activation.^47^ We previously have shown that CSN6 is a positive regulator of c-Myc,^31^ it remains to been seen whether CSN6 can enhance FASN gene expression through c-Myc although we have observed that CSN6 have no impact on FASN mRNA expression. mTORC1 can promote lipid biogenesis through SRPK2, a critical regulator of RNA-binding SR proteins, which in turn regulates RNA stability of lipid biosynthetic enzymes including FASN.^50^ Since AKT is the upstream of mTORC1, it is conceivable that AKT can also regulate the RNA stability of lipogenic enzymes. E3 ubiquitin ligase TRIM21 can mediate FASN ubiquitination,^51^ but this regulation requires acetylation catalyzed by KAT8. We have shown that CSN6 promotes ubiquitin-dependent degradation of TRIM21,^41^ and it is then possible that CSN6 can regulate FASN stability through TRIM21 as well. It has been shown that ubiquitin-specific protease 14 (USP14) can deubiquitinate FASN and increase its stability^52^ but the E3 ligase involved has not been characterized. Our studies regarding CSN6’s role in regulating FASN ubiquitination and stability may be involved TRIM21 or USP14 in addition to FBXW7β. It is interesting to further characterize the possible interaction of these molecules. AKT signaling activation is observed in a series of cancers, and it appears as a promising therapeutic target for many cancers. Although AKT is highly active in different advanced cancers, the biological significance of AKT targets’ aberrant regulation remains not well characterized. GSK3β is inhibited via AKT-mediated phosphorylation. Our data demonstrates that GSK3β catalyzes FASN phosphorylation, which destabilizes FASN via enhancing FBXW7β-mediated FASN ubiquitination and degradation. In summary, AKT activation can increase FASN stability to maintain continuous de novo lipogenesis.

FBXW7 exists in three splice-variant isoforms (α, β, γ) with specific subcellular localization. However, the role and the regulation of FBXW7 isoform expression have not been well characterized.^53^ For instance, FBXW7β can be induced by the stress stimuli in a p53-dependent manner while FBXW7α and γ seems to be p53-independent.^53^ It needs to be emphasized that the identified FBXW7β, a cytosolic form, is the most effective in affecting FASN degradation since FASN is also majorly located in the cytoplasm. As for AKT’s impact on lipogenesis, mTORC2, an activator of AKT, enhances de novo fatty acid synthesis in liver, leading to tumor malignancy.^7^ However, how mTORC2 regulates FA synthesis is not clearly understood.^7^ Our studies suggest that mTORC2 may activate AKT to cause FASN stabilization, thereby promoting de novo fatty acid synthesis. The observation that FBXW7β-mediated FASN degradation is reminiscent of the regulation of many other FBXW7 substrates. For examples, upon phosphorylation of c-MYC by GSK3β kinase, FBXW7 recognized and degraded c-MYC.^24^ FBXW7 is a tumor suppressor^54^ that recognizes and degrades a series of oncoproteins, such as c-MYC, c-JUN, Cyclin E, MTOR, Aurora B, Notch, MCL-1, FOXO4 and CHD6.^23–35^ Therefore, it makes sense that the tumor suppressive E3 ligase FBXW7β causes degradation of a potential oncoprotein FASN. Indeed, we characterized cancer-specific hot spot mutations of FBXW7β (R385H/C) on WD40 propeller substrate binding sites from CRC patients. The biological function of these mutants has not been characterized yet. We found that these cancer-derived FBXW7β mutants lost their activity toward FASN degradation and in inhibiting lipogenesis, indicating that its intact activity is responsible for tumor suppression. Our results add a new target member for FBXW7-associated E3 ligase. Few evidence is shown to unravel the role of FBXW7β in lipogenesis, and our studies about the FBXW7β-FASN link provide insights for the first time into the important lipogenic roles of this incredible tumor suppressor.

FASN inhibitor orlistat has been approved by FDA.^2^ Orlistat can inhibit the absorption of dietary fat and is used for obesity management. So far, its use in cancer therapy has not been described although our PDX experiment and other animal cancer models have demonstrated promising effect in controlling cancer growth. However, its effect seems to depend on the CSN6 overexpression status as low CSN6 CRC is resistant to the growth inhibitory effect in our PDX studies (Fig. 7f). PDX experiments demonstrate important proof-of concept in its use and will be one step closer for clinical trial. Other FASN inhibitors can be explored for the CSN6-overexpressing cancer as well, including IPI-9119,^55^ C75, EGCG, flavonoid^2^ and several other inhibitors TVB-2640.^56–58^ It will be an exciting avenue for using these inhibitors in cancer prevention or therapy; for examples, in CRC patients when FASN expression was prognostically detrimental^59^ or in breast cancer patients with metastasis.^60^

We found that EGF can elevate lipid accumulation in CRC cell line with concurrent upregulation of CSN6 and FASN. Since both EGF and CSN6 signaling pathways can promote FASN accumulation, it is reasonable to deduce that inhibiting EGF signaling by cetuximab treatment plus attenuating FASN activity by orlistat might synergistically suppress CSN6-high CRC growth. Indeed, combining cetuximab with orlistat as a targeted strategy for *RAS/RAF* WT and CSN6-high colorectal PDX showed a better treatment efficacy than cetuximab or orlistat alone (Fig. 8e, f). In other words, orlistat may potentiate the treatment efficacy of cetuximab. These results bear pivotal prognostic and therapeutic implications for improving therapeutic efficacy of those CRC patients without *KRAS* mutation. Especially, these finding provide insights into why not all *RAS/RAF* WT CRC patients benefit from cetuximab treatment (only about 50% of the patients respond well).^61^ These results highlighted that the status of other factors needs to be considered, such as CSN6 and FASN levels, before the administration of cetuximab.

In conclusion, our results demonstrate a relationship among CSN6 expression, FBXW7β ubiquitination, FASN stability, FA synthesis and tumorigenicity. The impact of CSN6 in regulating FASN stability via FBXW7β elucidates a new layer of regulation regarding the activation of FASN during tumorigenicity. Given that FBXW7β is a tumor suppressor, our finding that FBXW7β is a critical component of lipogenesis regulatory circuitry re-illustrates the link between lipogenesis and cancer. Further developing compounds that promote FBXW7β-mediated FASN degradation or inhibit CSN6/FASN activity can be further developed as a therapeutic design for CSN6-overexpressing cancers.

## Materials and methods

### Patients and tissue samples

Fresh frozen paired samples of primary CRC, adjacent normal colon tissue and liver metastasis tissue were collected from the Department of Surgery at the First Affiliated Hospital of Sun Yat-sen University. We also obtained paraffin-embedded specimens of primary CRC and adjacent normal tissue from the Department of Surgery at the Six Affiliated Hospital of Sun Yat-sen University. The human tissue microarrays (TMAs) were obtained from Shanghai Outdo Biotech Company (HColA180Su16, #XT17-024), including 76 paired specimens of colorectal cancer tissues and adjacent normal tissues and 27 identified colorectal cancer samples. The original immunostained slides were scanned by Panoramic MIDI (3DHISTECH) and analyzed by Aipathweel. Patient information was presented in Supplementary Tables 3–4. All tissue samples were collected with the patient informed consent, and center’s Institutional Review Board approved this study.

### Cell culture and transfection

All the cells were obtained from ATCC, confirmed mycoplasma-free by PCR, and cultured at 37 °C and 5% CO_2_. DLD-1, HCT-8, HCT-15 and SW480 cells were cultured in RPMI 1640 medium (RPMI 1640) (Corning) supplemented with 10% (v/v) fetal bovine serum (FBS) (GIBCO). HEK293T, U2OS, HCEC, HCT 116, HT-29, WiDr, SW620 and COLO205 cells were cultured with Dulbecco’s modified Eagle’s medium (DMEM) (Corning) with 10% FBS. RKO were cultured with Minimum Essential Medium (MEM) (GIBCO) with 10% FBS.

For transient transfection, cells were seeded at a 60–70% confluency and allowed to attach overnight. Transfection was carried out using Polyethylenimine MAX 40,000 (Polyscience, 24765) or Lipofectamine 2000 (Invitrogen) following the instructions of manufacturer. The efficiency of transfection was verified by western blotting.

### DNA constructs and mutagenesis

Human CSN6 was cloned into pCMV5-Flag, pCMV5-HA, pCMV5-Myc, pCDNA3.1-Myc or pLVX vector using ClonExpress®II One Step Cloning Kit (Vazyme). Three isoforms of human FBXW7 were cloned into pCDNA3.1-Flag, pCDNA3.1-HA, pCDNA3.1-Myc or pLVX vector. Human pCDNA3.1-Flag-FASN was kindly provided by Dr Yue Xiong’s lab and cloned into pCDNA3.1-HA and pCDNA3.1-Myc. FASN T827A/S831A, FASN T827D/S831D, FBXW7β R385C, R385H, R399Q, R425C and R425H were made following the standard protocol of Mut Express II Fast Mutagenesis Kit V2 (Vazyme). The sequences of the mutagenic primer are listed in Supplementary Table 5. CSN6-N-terminal, CSN6-C-terminal, FBXW7β F-box domain deletion and FBXW7β WD40 domain deletion were constructed using ClonExpress®II One Step Cloning Kit (Vazyme) as well. The rest of constructs were obtained from our own institute. The resulting plasmids were sequence verified.

### Generation of stable transfectants

For the generation of CSN6 knock-down cell lines, 1 × 10^7^ HEK293T cells were seeded in 10-cm dish at a 70–80% confluency and allowed to attach overnight. Transfection was carried out using Polyethylenimine MAX 40,000 (Polyscience, 24765) with 10 μg pGIPZ-sh*CSN6*#1 or pGIPZ-sh*CSN6*#2 or corresponding vectors (pGIPZ-scramble shRNA), package vectors 5 μg psPAX2 and 5 μg pMD2.G. The supernatant was collected every 24 h for 48 h after initial transfection using filter Millex-GP Filter Unit (0.45 μm pore size, Millipore). For infection, cells were grown at 40–50% confluence and infected with 2 mL indicated viral supernatant, 200 μL FBS and 10 μg/mL polybrene (Millipore, TR-1003-G). The efficiency of knockdown was verified by western blotting.

For the generation of CSN6/FBXW7β dox inducible knock-down cell lines, lentiviral particles were packaged by co-transfection with 10 μg pLKO.1-puro (Plasmid 8453; Addgene) sh*CSN6*#1/#2 or sh*FBXW7β*, 5 μg psPAX2 and 5 μg pMD2.G in HEK293T cells. Infected cells were selected by puromycin (Selleck, S7417) for 1 week and then treated with or without doxycycline (Selleck, S4163) for indicated times. The knockdown efficiency was verified by western blotting.

The targeting sequences were as follows:

scramble shRNA: CTTCTAACACCGGAGGTCTT;

sh*CSN6*-#1: CTTGAGAGAAACCGCTGTCAT;

sh*CSN6*-#2: CAGTTTGTGAACAAGTTCAAT.

sh*FBXW7β*: ATGGGTTTCTACGGCACATTA

For the generation of CSN6/FBXW7β overexpression cell lines, lentiviral particles were generated by co-transfecting HEK293T cells with 10 μg pLVX-CSN6, pLVX-FBXW7β or corresponding vectors (plvx-vector), 5 μg psPAX2 and 5 μg pMD2.G. The overexpression efficiency was verified by western blotting.

### Cell proliferation analysis

The growth of colorectal cancer cells was analyzed using IncuCyte ZOOM Live-Cell Analysis System (Essen BioScience, MI, USA), which allows real-time quantitative detection of live cells. The cells were settled at 37 °C and 5% CO_2_ overnight. When a confluence of 10–20% was reached, indicated treatment was added to the proper wells. Thereafter, cells were placed in the IncuCyte at 37 °C and 5% CO_2_ for monitoring. Images were taken of each well at 4× magnification every 2 h. Data processing was performed using Zoom2016 A software (Essen BioScience).

### Cell viability assay

Drug treatment efficiency of cells was determined by Cell Counting Kit-8 (ApexBio Technology) following the standard protocol. In brief, 2000 cells were seeded in 96-well plates and settled at 37 °C and 5% CO_2_ overnight. Gradient concentration of drugs was added for 72 h. Where indicated, cells were incubated in 100 μL 10% (v/v) CCK8/complete medium mixture for 1–4 h at 37 °C and 5% CO_2_. Cell viability was monitored by absorbance 450 using microplate reader.

### Colony formation assay

200 cells were seeded into 12-well plates and settled at 37 °C and 5% CO_2_ for 3 days. Cells were treated with drugs or the drugs vehicle alone for two weeks, followed by fixing with 4% formaldehyde for 5 min, permeabilizing in methanol for 20 min and staining with 0.5% crystal violet for 20 min, washing and analyzing. Quantification was performed using Fiji. Three independent experiments were performed.

### Transwell migration assay

For migration assay, 1 × 10^5^ cells suspended in 150 μL serum-free medium were seeded into 24-well Boyden chambers (Corning, NY) and 750 μL medium with 10% FBS was placed in the lower chamber as a chemoattractant. 18 h later, cells were fixed with 4% formaldehyde for 5 min, permeabilized in methanol for 20 min and stained with 0.5% crystal violet for 20 min. Swabbed the insert of each chamber and counted under microscope. The average number of cells under each microscopic field was calculated for over three areas per filter. Experiments were performed at least three times.

### Orlistat preparation

For in vitro experiments, orlistat (sigma, O4139) was prepared as a 19 mg/mL stock solution by dissolving in 100% (v/v) DMSO. For in vivo studies, orlistat was prepared following the methods reported by Kridel et al.^62^ In brief, orlistat (aladdin, O159936) was dissolved in 33% (v/v) ethanol and 66% (v/v) PEG400 for 30 min at room temperature, vortexed every 10 min. The solution (48 mg/mL orlistat) was aliquoted and stored at −80 °C.

### Palmitate solution preparation and treatment

Palmitate (aladdin, P101058) was prepared as a 10 mM stock solution by dissolving it in 100% (v/v) ethanol. The stock solution was aliquoted and stored at −80 °C. For palmitate rescue experiments, CRC cells growing in medium containing regular FBS were treated in vitro with 200 μM palmitate or 2% (v/v) ethanol (vehicle) for 48 h.

### CHIR-99021 solution preparation and treatment

For in vitro experiments, CHIR-99021 (selleck, S2924) was prepared as a 10 mM stock solution. The stock solution was aliquoted and stored at −80 °C. For in vivo studies, CHIR-99021 (aladdin, C125082) was solubilized in 4% (v/v) DMSO and 30% (v/v) PEG300. The solution (3 mg/mL CHIR-99021) was aliquoted and stored at −80 °C.

### Immunoblotting and immunoprecipitation

For immunoblotting, cells were collected and lysed in ice-cold lysis buffer (50 mM Tris–HCl (pH 7.5), 0.1% TritonX-100, 1 mM EDTA, 150 mM NaCl, 0.1% Nonidet P-40) with fresh protease inhibitor cocktail and phosphatase inhibitor cocktail (Bimake). Lysates were sonicated, incubated on ice for 30 min and then centrifugated. Total protein concentration was assessed by a BCA protein assay kit (BestBio). For tissue samples, proper amount of the tissues (as large as a rice) was homogenized using Tissuelyser in ice-cold lysis buffer. Lysates were collected following centrifugation at 12,000 × *g* for 15 min at 4 °C. The protein supernatants were prepared as before. Equal amounts of total protein were loaded on SDS-PAGE gel and then transferred onto polyvinylidene difluoride (PVDF) membranes (Millipore). The membranes were blocked in 5% skim milk in 1xTBST buffer at room temperature for 1 h and incubated in indicated primary antibodies overnight at 4 °C, followed by incubating with secondary horseradish peroxidase-conjugated goat anti-mouse or anti-rabbit antibodies (Thermo) for 1 h at room temperature. Chemiluminescent images of the membranes with immunodetected bands were detected by ChemiDoc™ Touch (BIORAD) using Clarity™ Western ECL Substrate (BIORAD). The expression of Tubulin, GAPDH or Vinculin were used as loading control.

For in vivo immunoprecipitation, cells were treated with 20 μM MG132 (Selleck, S2619) 6 h before harvest, after which they were lysed for 30 min on ice in cell lysis buffer and centrifugated. 10% of supernatants were used as input. For endogenous immunoprecipitation, cell lysates were incubated with endogenous antibody or control IgG (Sigma, I5006) overnight at 4 °C, followed by incubating with Protein A/G agarose beads (Santa cruz) for 4 h at 4 °C. For Flag-immunoprecipitation, cell lysates were incubated with anti-Flag M2 agarose beads (Sigma, A2220) overnight at 4 °C. For Myc-immunoprecipitation, cell lysates were incubated with anti-Myc magnetic beads (Biotool, B26302) overnight at 4 °C. The beads were then washed with ice-cold lysis buffer five times and boiled in 2 × SDS loading buffer for 10 min.

For in vitro immunoprecipitation, proteins were produced with TNT Quick Coupled Transcription/Translation systems (Promega, L1170) according to the manufacturer’s instructions. The indicated proteins were pulled down with anti-Flag M2 beads or anti-Myc beads and immunoblotted with the indicated antibodies.

### Antibody

The antibodies we used in immunoblotting were as follows: CSN6 1:4000 (Enzo Life Sciences, BML-PW8295), FASN 1:4000 (Cell Signaling Technology, 3180S), p-AKT 1:1000 (Cell Signaling Technology, 4060S), AKT 1:2000 (Cell Signaling Technology, 2920S), p-ERK1/2 1:4000 (Cell Signaling Technology, 4370), ERK1/2 1:4000 (Cell Signaling Technology, 4695S), FBXW7 1:5000 (Abcam, ab109617), Flag-Tag 1:5000 (Sigma, F1804), HA-Tag 1:5000 (Proteintech, 51064-2-AP), Myc-Tag 1:5000 (Cell Signaling Technology, 2276S), β-Catenin 1:4000 (BD Biosciences, 610153), GSK-3-β 1:4000 (Cell Signaling Technology, 9832S), GAPDH 1:5000 (Proteintech, 60004-1-Ig), alpha Tubulin 1:5000 (Proteintech, 66031-1-Ig), Vinculin 1:4000 (Cell Signaling Technology, 4650s), Lamin B1 1:5000 (abcam), PARP-1 1:4000 (Cell Signaling Technology, 9532S), Phospho-Threonine (Cell Signaling Technology, 9386s).

The antibodies we used in immunohistochemistry were as follows: CSN6 (Enzo Life Sciences, BML-PW8295), FASN (Cell Signaling Technology, 3180S), Ki67 (Proteintech, 27309-1-AP) and Cleaved caspase-3 (Cell Signaling Technology, 9664P).

### Ubiquitination assay

For His ubiquitination assay, cells were co-transfected with the indicated plasmids for 48 h, followed by treatment with 20 μM MG132 (Selleck, S2619) for 6 h. Cells were collected and subjected to lyse in Buffer A (6 M guanidine-HCl, 10 mM imidazole, 0.1 M Na_2_HPO_4_/NaH_2_PO_4_; pH 8.0). The ubiquitinated proteins were pulled down with Ni-NTA agarose beads (Invitrogen, R901-15) for 4 h at room temperature, washed twice with Buffer A, followed by washed twice with Buffer A/TI (1:3, v/v), after which washed once with Buffer TI (25 mM Tris-HCl, 20 mM imidazole; pH 6.0) and boiled in 1 × SDS loading buffer for 10 min. The ubiquitinated proteins were detected with the indicated antibodies.

For HA ubiquitination assay, cells were prepared and lysed in ice-cold lysis buffer for 30 min and centrifugated. 10% of supernatants were used as input. The remaining supernatants were incubated with anti-Flag M2 agarose beads or anti-Myc magnetic beads for overnight at 4 °C. The beads were then washed five times with ice-cold lysis buffer and boiled in 2 × SDS loading buffer for 10 min. The ubiquitinated proteins were detected with anti-HA antibody.

### Turnover assay

The cells were prepared and seeded in six-well plates at 80% confluency. Cycloheximide (CHX) (Sigma) was added at a concentration of 20 μg/mL. Cells were then harvested at different time points and analyzed by western blotting.

### Immunohistochemistry (IHC)

Briefly, paraffin-embedded sections (4 μm) were dewaxed in 100% (v/v) xylene and rehydrated in descending concentrations of ethanol. Antigen retrieval were performed using 1 mM EDTA unmasking solution (ZSGB-BIO, ZLI-9069) by microwave heating. After cool down, sections were incubated in 3% hydrogen peroxide for 30 min in dark at room temperature, blocked with goat serum (ZSGB-BIO, ZLI-9056) for 1 h at room temperature, and probed with corresponding primary antibody at 4 °C overnight. Afterward, the sections were incubated with biotinylated goat anti-rabbit/mouse IgG (ZSGB-BIO, PV-6000) for 15 min at room temperature. Immunostaining was visualized with diaminobenzidine (DAB) (ZSGB-BIO, ZLI-9018), and sections were then counterstained with hematoxylin (Servicebio, G1004) and mounted with cover slip using neutral resins.

### Immunofluorescence (IF)

For tissue IF staining, frozen-slides were prepared and baked in 37 °C for 10–20 min to control the moisture, follow by fixed in 4% paraformaldehyde (PFA) for 30 min. Antigen retrieval were performed using 1 mM EDTA unmasking solution (ZSGB-BIO, ZLI-9069) by microwave heating. After cool down, slides were blocked with PBS/ 5% BSA for 1 h, and probed with indicated primary antibody diluted in PBS/ 1% BSA at 4 °C overnight. Afterward, the slides were incubated with Alexa Fluor-conjugated secondary antibodies (Invitrogen) at room temperature in dark for 1 h and then counterstained with 4,6-diamidino-2-phenylindole (DAPI, 5 μg/mL) for 10 min, kept in dark place. Stained sections were mounted with anti-fade mounting medium and images were taken using fluorescence microscope (Leica).

For cell IF staining, cells cultured on glass bottom cell culture dish (NEST, 801002) were fixed in 4% PFA for 15 min and washed three times with 1× PBS. The cells were then incubated with blocking buffer (1× PBS/5% BSA/0.3% Triton X-100) for 1 h at room temperature and probed with indicated primary antibody overnight at 4 °C, followed by incubating with Alexa Fluor-conjugated secondary antibodies (Invitrogen) at room temperature in the dark for 1 h. DAPI nuclear counterstaining was used. Images were taken by Leica TCS SP8 confocal laser scanning microscope.

### RNA extraction and quantitative reverse transcriptase PCR

Total RNA was extracted from cultured cells using the TRIzol Reagent (Invitrogen, #15596026), and reverse transcription were performed using ReverTra Ace® qPCR RT Master Mix with gDNA Remover (TOYOBO) following the standard protocols. Real-time PCR was performed using a LightCycler® 480 II (Roche) instrument with 2× SYBR Green qPCR Master Mix (biotool, #B21203). Primer sequences are listed in Supplementary Table 6. GAPDH was used as internal control. Triplicate reactions were carried out for each sample.

### Measurement of cellular lipid content

The lipophilic fluorescence dye BODIPY 493/503 (Molecular Probes, D3922) was used to monitor the content of neutral lipids in CRC cells.

For flow cytometry (FACS) analysis, CRC cells were rinsed with PBS, followed by incubation on 2 μM BODIPY working solution in the dark for 30 min at 37 °C. After a quick rinse with PBS, cells were trypsinized and prepared for FACS analysis. All samples were filtered through a 50 μm filter to get a single cell suspension. BODIPY positive cells were detected by CytoFLEX (BECKMAN COULTER, A00-1-1102). Analysis was performed using FlowJo software.

For confocal imaging of lipid droplet in CRC cell lines, cells cultured on glass bottom cell culture dish (NEST, 801001) were washed three times with 1× PBS, followed by incubating with 2 μM BODIPY working solution in the dark for 30 min at 37 °C. Staining was stopped by washing cells with 1× PBS and then fixed immediately with 4% (v/v) PFA for 30 min. Nuclei were counterstained by DAPI solution (5 μg/mL) for 10 min. Images were obtained by Leica TCS SP8 confocal laser scanning microscope.

For frozen tissue sections, slides were air-dried at room temperature for 10 min. 5 uM BODIPY working solution was applied to cross sections for 30 minutes at 37 °C. The sections were then washed 3 times with 1× PBS before fixation with 4% (v/v) PFA for 5 min. Following fixation, sections were washed 3 times in PBS for 5 min. DAPI (5 μg/mL) was used to identify nuclei. All samples were mounted with anti-fade mounting medium and covered with glass cover slips. Images were obtained using fluorescence microscope (Leica). Size and number of lipid droplet were quantified using Fiji.

### Frozen sections

Fresh mouse tumor tissues were dissected and immediately sealed in Optimal Cutting Temperature (O.C.T.) compound (Leica, 14020108926) by liquid nitrogen frozen. The sections were obtained by cutting tissues into 10-μm-thick using a cryostat microtome and stored at −80 °C.

### Subcellular fractionation

Cells (1 × 10^7^) were collected and resuspended in Fractionation Buffer (50 mM HEPES, pH 7.4, 150 mM NaCl, 1 mM EDTA, 1 mM sodium orthovanadate, protease inhibitor cocktail) with 0.2% NonidetP-40 for 5 min on ice. 5% of cells was used as the whole-cell lysate fraction. The rest of cells were centrifuged at 1500 r.p.m. for 5 min at 4 °C. The supernatant was harvested as cytosolic fraction, while the pellet was resuspended in Fractionation Buffer with 0.5% NonidetP-40, used as the nuclear fraction.

### Mouse models

All mouse experiments were approved by the Animal Ethical and Welfare Committee of Sun Yat-sen University and complied with institutional animal care guidelines. For injection mouse models, mice were randomized before being injected with cancer cells or compounds.

#### Subcutaneous DLD-1 xenograft model

2 × 10^6^ DLD-1 cells were injected subcutaneously into the right hind flank of 6-week-old female athymic nude mice. The treatment was initiated when tumor become palpable (40 mm^3^). For orlistat treatment, mice were either treated with vehicle (33% v/v ethanol, 66% v/v PEG400) alone or with 240 mg/kg FASN inhibitor orlistat once per day by intraperitoneal (i.p.) injection. For CHIR-99021 treatment, mice were either treated with vehicle (4% v/v DMSO, 30% v/v PEG300) alone or with 30 mg/kg GSK3β inhibitor CHIR-99021 every other day by oral gavage. Tumors growth were measured with calipers every three days and calculated according to the formula (length × width^2^) /2. The body weight was monitored since treatment. Humane endpoints were defined when tumor size reaching 1.5 cm^3^. For bioluminescence imaging, mice received 150 mg/kg D-luciferin (i.p.) were anesthetized using pentobarbital sodium (40 mg/kg) and photoed using IVIS Spectrum (Perkin-Elmer). ROIs of the displayed images were drawn around the tumor sites and quantified as photons/s using the Living Image software. The mice were then euthanized, and the tumors were collected and snap-frozen for further analyses. The number of ethical approval was 20190614-001.

#### Patient-derived xenograft (PDX) model

PDX mouse models were established as previously described.^44,45^ Primary tumor specimens were collected from colorectal cancer patients at the Sixth Affiliated Hospital of Sun Yat-sen University (Guangzhou, China). 6-week-old female NCG mice (GemPharmatech Co., Ltd) were used for tumor transplantation. In our studies, the xenograft tumors were 2–3 passage. Briefly, a small incision was made on the nape of anaesthetized NCG mice. Fresh colorectal tumor specimens were cut into 3 mm^3^ fragments and subcutaneously xenografted to the right hind flanks of NCG mice. Then the incision was sutured. The treatment was initiated when tumor become palpable (40 mm^3^). For single drug treatment, CSN6-low PDX line and CSN6-high PDX line were selected from 12 candidates by detecting CSN6 expression in tumor lysate by immunoblot. Mice were either treated with vehicle (33% v/v ethanol, 66% v/v PEG400) alone or with 240 mg/kg FASN inhibitor orlistat once per day by i.p. injection. The tumor formation and body weight of mice were monitored since treatment. Humane endpoints were defined when tumor size reaching 1.5 cm^3^. For the drug combination treatment, patient cases were selected with *KRAS/BRAF/NRAS* wild type and CSN6 high level. The mice were separated into four groups: vehicle control, cetuximab (40 mg/kg, Selleck) alone, orlistat (240 mg/kg, aladdin) alone, cetuximab and orlistat combination. The study of ethical approval was IACUC-2020122905.

### In vivo fatty acid measurement

At the end of mouse experiment, mice were starved overnight and then anesthetized using pentobarbital sodium (40 mg/kg). Mice were intraperitoneally (i.p.) injected with a ^13^C-glucose solution (2 g/kg in PBS). 2.5 h later, mice were euthanized and tumors were dissected. Proper amount of the tissues (as large as a rice) was added to 400 μL of cold extraction solution (80% (v/v) HPLC-grade methanol) and then homogenized using TISSUELYSER (JINGXIN TECHNOLOGY, JXFSTPRP-32). After centrifugation the supernatant was moved to new tubes for dryness and used for fatty acid analysis.

#### Patient-derived organoids (PDOs) culture

Patient-derived organoids culture of colorectal cancer was performed as previously described.^42,43^ Fresh tumor tissues collected from CRC patients were washed three times with ice-cold PBS containing penicillin-streptomycin, and minced into small pieces (3–5 mm) quickly, then digested with EDTA (5 mM) for 60 min on ice. When the cells were in clumps under microscopic observations, finished digestion. The cells were mixed with Matrigel (Corning) and then seeded into a 24-well plate. When Matrigel polymerisation (10 min at 37 °C), added 500 μL advanced DMEM/F12 medium containing 10 mM HEPES, 1× N2 (Life Technologies), 2 mM GlutaMAX, 1× B27, 10 nM gastrin I (Biogems), 50 ng/mL recombinant EGF, 500 nM A83-01 (Biogems), 100 ng/mL recombinant Noggin (Peprotech), 500 ng/mL R-spondin-1 (Peprotech), 10 μM SB202190 (Sigma), 10 μM Y-27632 (Abmole), 10 mM nicotinamide (Sigma), 1 mM N-acetylcysteine (Sigma) and 100 U/mL penicillin/streptomycin. 24 h later, the organoids were infected with a non-targeting hairpin or sh*CSN6* lentivirus using polybrene (10 μg/mL, Millipore, TR-1003-G) for 6 hours, then replaced the medium with fresh medium and renewed every three days. The organoid images were taken at day 0, 3, 6, 9. The size and number of organoids were quantified using Fiji.

### Statistical analysis

Each experiment was performed at least three times. Data were presented as mean ± SD. Differences among two groups were assessed by two-tailed Student’s *t* test. Multiple group comparisons were analyzed by One-way ANOVA. Paired samples were analyzed using a paired *t* test. For cell or tumor growth curve, two-way ANOVA analysis was applied. For survival analysis of colorectal cancer patients, Kaplan–Meier survival curves and log-rank test were used. A *p* value of less than 0.05 was defined as statistically significant (*P < 0.05, **P < 0.01, ***P < 0.001, ****P < 0.0001). Details on specific statistical tests can be found in each figure legend. All of the statistical analyses were performed using SPSS 25.0 and GraphPad Prism 8.0 software.

### Bioinformatics analysis

Expression data of FASN and CSN6 protein in colorectal cancer was downloaded from The Cancer Proteome Atlas (TCPA; https://tcpaportal.org/tcpa/) and Clinical Proteomic Tumor Analysis Consortium database (CPTAC; https://proteomics.cancer.gov/programs/cptac).

### Untargeted lipidomics

HCT 116 cells with scramble shRNA and sh*CSN6* were harvested in 5 mL Vacutainer tubes containing the chelating agent EDTA, and centrifuged at 1500 × *g* for 15 min at 4 °C. The supernatants were aliquoted and stored at −80 °C until LC-MS/MS analysis. Lipids were extracted according to MTBE method. In brief, samples were thawed at 4 °C and homogenized with cold methanol/water (1:1, v/v). After adding 800 μL of MTBE, the mixture was ultrasonic for 20 minutes at 4 °C, followed by centrifugation at 14,000 × *g* for 15 min at 10 °C. The organic layer was obtained and dried under nitrogen. For LC-MS analysis, the collected samples were redissolved in 200 μL 90% isopropanol/ acetonitrile. Analysis was performed using an UHPLC Nexera LC-30A coupled to Q Executive plus mass spectra (Thermo Scientific™) in Shanghai Applied Protein Technology Co., Ltd. For data processing, LipidSearch software version 4.1 (Thermo ScientificTM) was used to identify and quantify the lipid species. Multivariate data analysis, including Pareto-scaled principal component analysis (PCA), partial least squares discrimination analysis (PLS-DA) and orthogonal partial least-squares discriminant analysis (OPLS-DA) were applied to analyze the processed data. Student’s *t*-test was used to assess the significance of each metabolite at univariate level. *P* values less than 0.05 were defined as statistically significant.

### Gel filtration

The cell fraction assay was performed as previously described.^63^ Briefly, 10-cm dish of HCT-8 and HCT 116 cells were lysed to extract proteins as described before. Cellular extracts prepared with 5 mg protein/500 μL lysis buffer were subject to a Millipore Ultra free centrifugal filter apparatus (3 kDa nominal molecular mass limit) and then applied to an 850*20 mm Superose 6 size exclusion column (Amersham Biosciences) that had been equilibrated with buffer A-150. To define the eluant positions of protein with known molecular mass (kDa), calibrated protein standards (blue dextran, 2000 kDa; thyroglobulin, 669 kDa; ferritin, 440 kDa; bovine gamma globulin, 158 kDa; bovine serum albumin, 75 kDa) were used. Fractions were collected for further analysis by western blotting.

## Supplementary information

Supplementary Materials

## Data Availability

The data used in the current study are available from the corresponding authors upon reasonable request.
