# Supplementary material for: FBXW7β loss-of-function enhances FASN-mediated lipogenesis and promotes colorectal cancer growth
Source: Signal Transduct Target Ther. 2023 May 19;8:187. doi: 10.1038/s41392-023-01405-8 (PMC10195794; doi:10.1038/s41392-023-01405-8)
Supplement: Supplementary file 1 — Supplementary Materials [file 41392_2023_1405_MOESM1_ESM.docx]

Supplementary Materials for

**FBXW7β loss-of-function enhances FASN-mediated lipogenesis and promotes colorectal cancer growth**

Wenxia Wei^1,2^*, Baifu Qin^1,2^*, Weijie Wen^1,2^*, Boyu Zhang^1,2^, Haidan Luo^1,2^, Yuzhi Wang^1,2^, Hui Xu^1,2^, Xiaoshan Xie^1,2^, Sicheng Liu^2,5^, Xin Jiang^1,2^, Mengan Wang^1,2^, Qin Tang^6^, Runxiang Yang^5^, Zongmin Fan^1,2^, Haiwen Lyu^1,2^, Jiayu Zhang^1,2^, Junzhong Lin^4^, Kai Li^1,2 ‡^, and Mong-Hong Lee^1,2,3 ‡^

^1^Guangdong Provincial Key laboratory of Colorectal and Pelvic Floor Disease, The Sixth Affiliated Hospital, Sun Yat-sen University, Guangzhou 510655, China

^2^Guangdong Institute of Gastroenterology, Guangzhou 510655, China

^3^Department of Oncology, The Sixth Affiliated Hospital, Sun Yat-sen University, Guangzhou 510655, China

^4^Department of Colorectal Surgery, Cancer Center, Sun Yat-sen University, Guangzhou 510060, China

^5^Second Department of Medical Oncology, The Third Affiliated Hospital of Kunming Medical University, Kunming, 650118, China

^6^Guangzhou Institute of Pediatrics, Guangzhou Women and Children’s Medical Center, Guangzhou Medical University, Guangzhou, 510623, China.

*These authors contributed equally to this work.

‡Correspondence to Mong-Hong Lee (limh33@mail.sysu.edu.cn), Kai Li (likai39@mail.sysu.edu.cn)

**This PDF file includes:**

Figures. S1 to S7

Tables S1 to S6

Figure. S1.


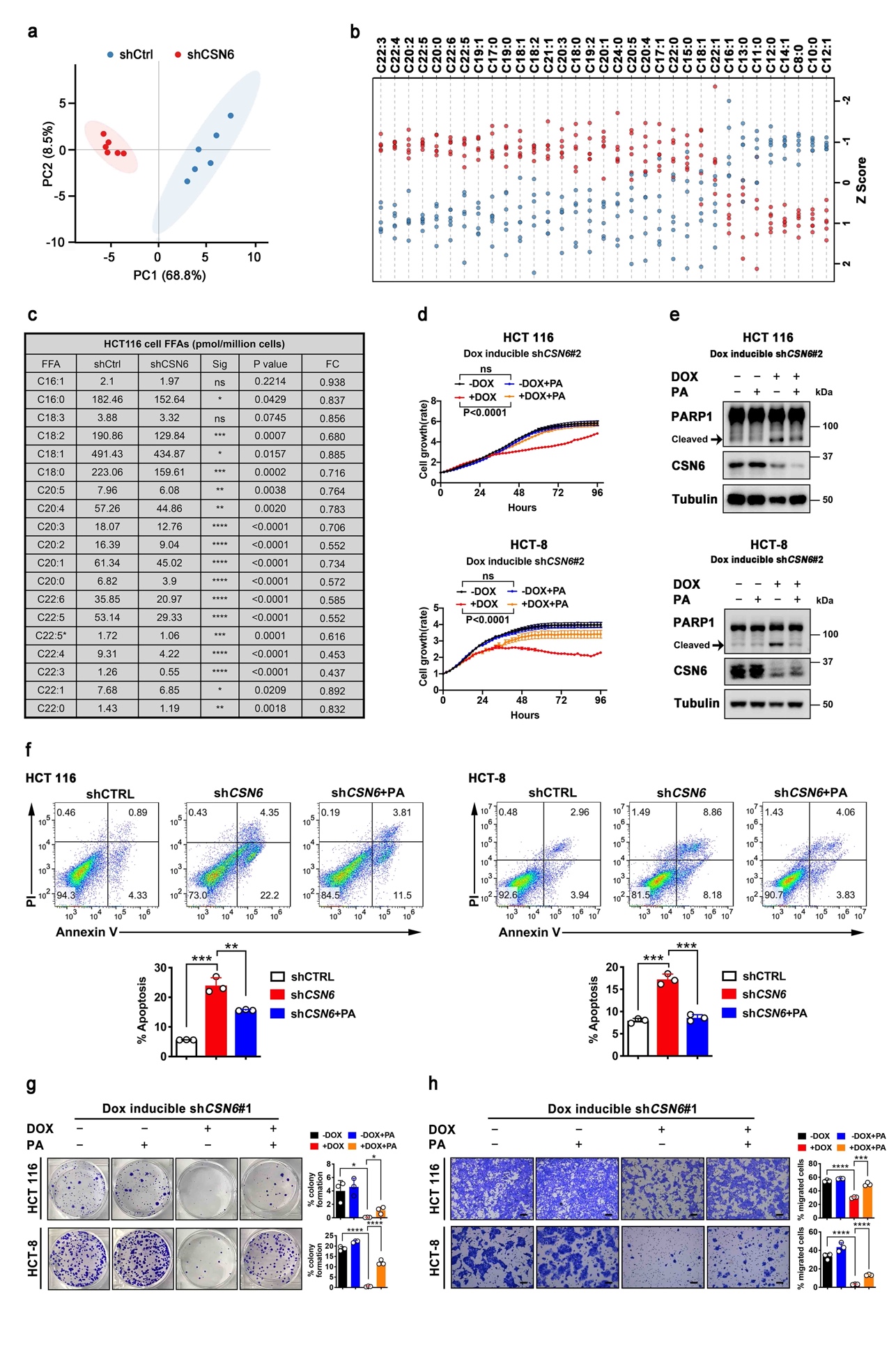


**Figure. S1. Deregulation of CSN6 reprograms lipid biosynthesis in CRC. (**a) CSN6 KD revealed a marked reduction in the pools of a number of free fatty acids (FFAs). Principal-component analysis (PCA) of targeted lipidomic analysis from HCT116 cells infected with control shRNA or CSN6 shRNA (*n*=6 per group). PC1 and PC2 explain 68.8% and 8.5% of the variation, respectively. **(**b) Changes of metabolites were determined by mass spectrometry in HCT116 cells infected with control shRNA or CSN6 shRNA (*n*=6 per group). Each circle is an individual sample. **(**c) Table showing levels of free fatty acids (FFA) in HCT116 cells infected with control shRNA or CSN6 shRNA (*n*=6 per group). Table shows names of FFA, average level (pmol/million cells) in shCtrl or sh*CSN6* cells, significance, p-value and fold change (FC) (vs. shCtrl). ns not significant, **P* < 0.05, ***P* < 0.01, *****P* < 0.0001; as determined by two-sided Student’s t-test. **(**d) CSN6 KD led to cell growth inhibition. Palmitate attenuated the impact of CSN6 KD. Cell growth assay generated from dox inducible sh*CSN6*#2 HCT116 (upper) and HCT8 (bottom) cells pretreated with or without doxycycline (DOX, 200 μg/mL) for 48 hours and then treated with ± 200 μM exogenous palmitate (PA) in medium containing regular FBS. Representative data of triplicate experiments are shown. Statistical significance of result was tested using Two-way ANOVA, ns not significant. **(**e) CSN6 KD led to cell apoptosis. Palmitate attenuated the impact of CSN6 KD. Immunoblot analysis of apoptosis marker Cleaved PARP1 in dox inducible sh*CSN6*#2 HCT116 and HCT8 cells pretreated with or without doxycycline (DOX, 200 μg/mL) for 48 hours and then treated with ± 200 μM exogenous palmitate (PA) in medium containing regular FBS. Arrow denotes molecular weight of Cleaved PARP1. **(**f) Cell apoptosis assay for HCT116 cells (left) and HCT8 cells (right) infected with control shRNA or CSN6 shRNA and then treated with ± 200 μM exogenous palmitate (PA) in medium containing regular FBS. Representative data of triplicate experiments are shown. Statistical significance of result was tested using one-way ANOVA. Data are represented as mean ± SD, ***P* < 0.01, ****P* < 0.001. **(**g-h) CSN6 KD impacts on colony formation and cell migration. Palmitate attenuated the impact of CSN6 KD. Colony-formation assays (g) and migration assays (h) generated from dox inducible sh*CSN6*#1 HCT116 and HCT8 cells pretreated with or without doxycycline (DOX, 200 μg/mL) for 48 hours and then treated with± 200 μM exogenous palmitate (PA) in medium containing regular FBS. Representative data of triplicate experiments are shown. Scale bar, 100 μm. Statistical significance of result was tested using one-way ANOVA. Data are represented as mean ± SD, **P* < 0.05, ****P* < 0.001, *****P* < 0.0001.

Figure. S2.


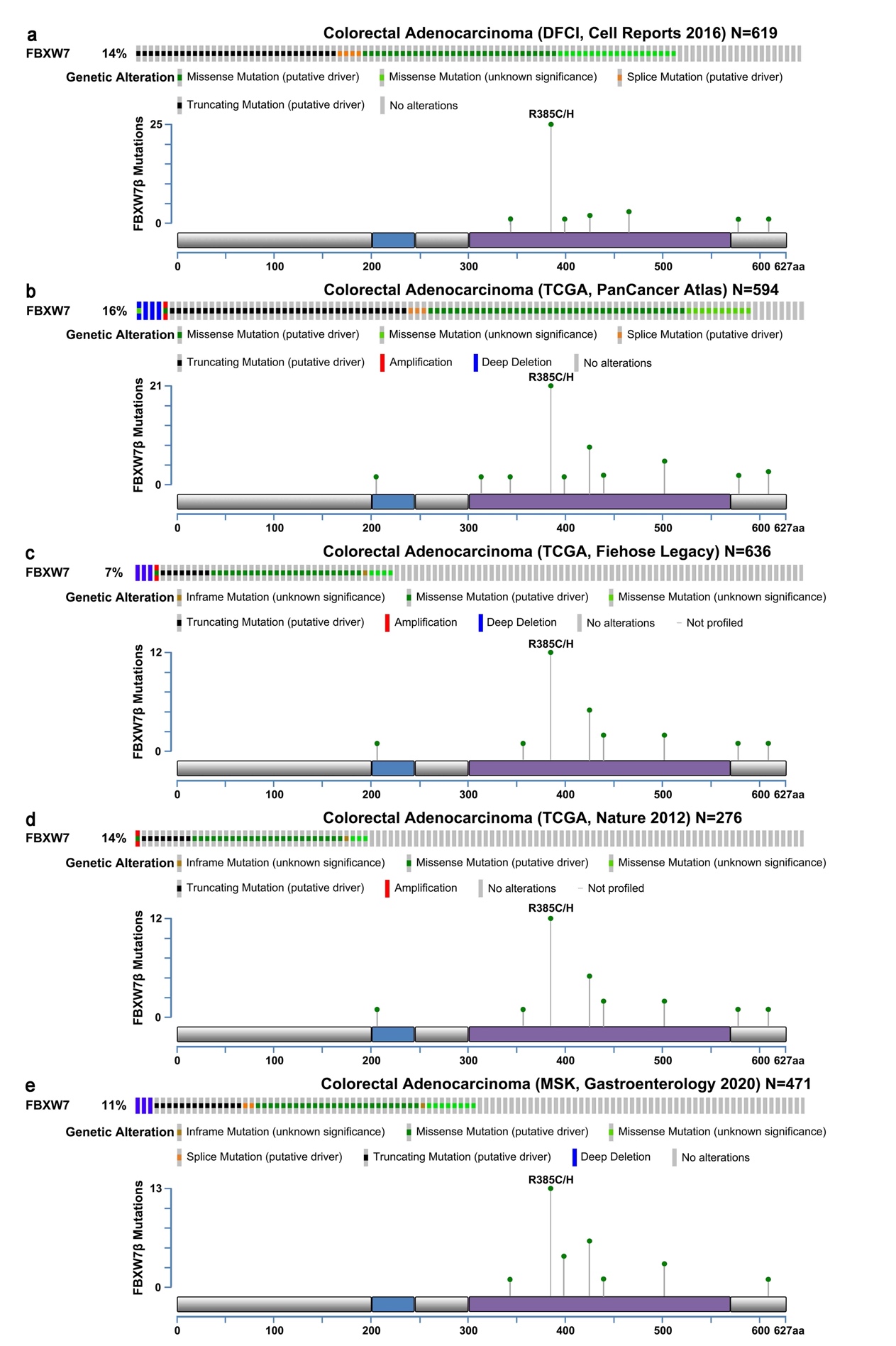


**Figure. S2. FBXW7β hot spot mutations in CRC.** (a-e) Genetic alterations of FBXW7β in colorectal adenocarcinoma database (<http://www.cbioportal.org/datasets>). The gene alteration percentages are shown. Missense mutations (putative driver) diagram of FBXW7β were shown. Each circle is determined with respect to the mutation site and case number. The most frequent mutation of FBXW7β is R385H/C.

Figure. S3.


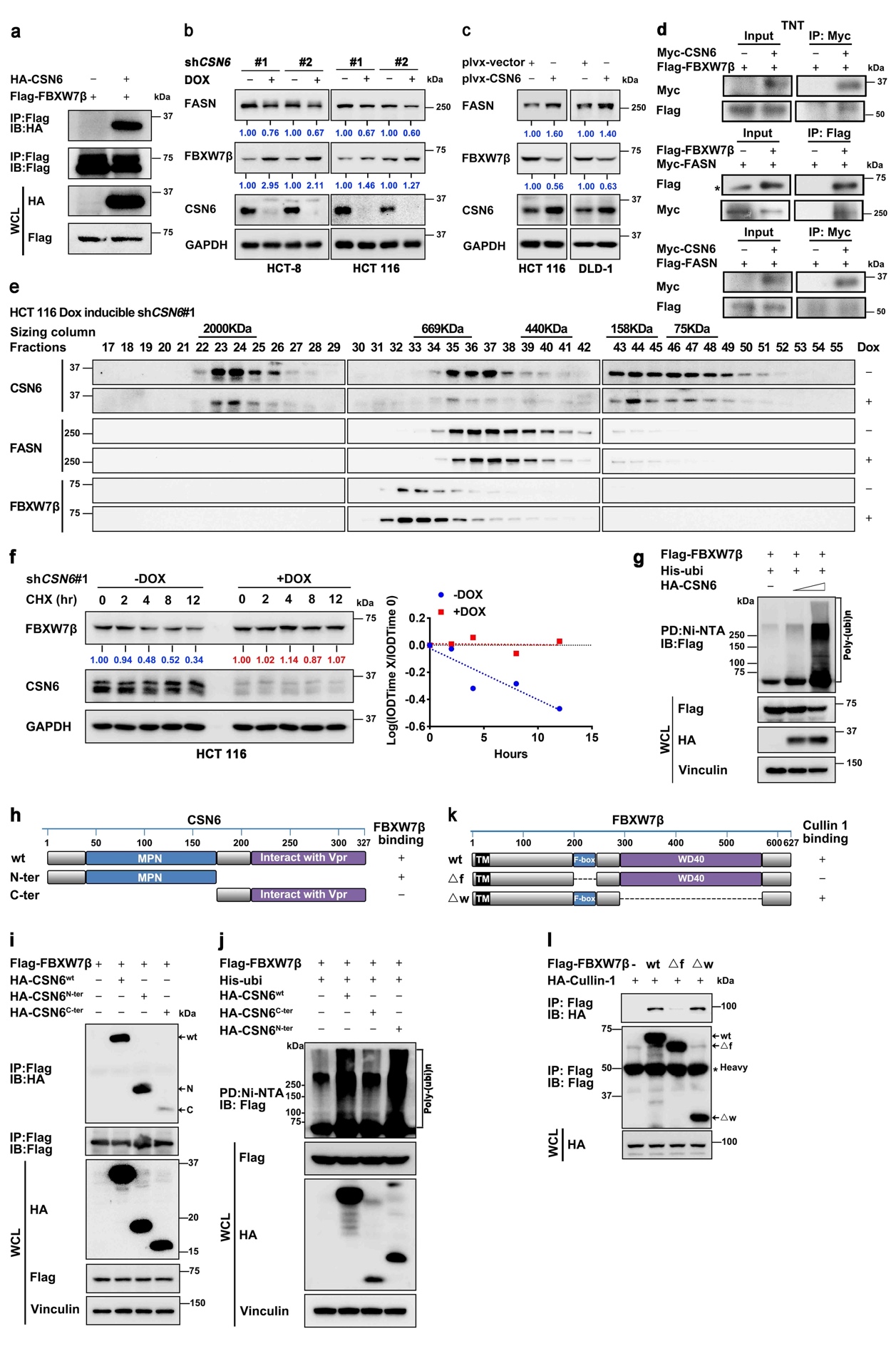


**Figure. S3. CSN6 enhances FBXW7β autoubiquitination and degradation through physical interaction.** (a) Exogenous interaction between CSN6 and FBXW7β. CSN6 interacts with FBXW7β. HEK293T cells expressing Flag-FBXW7β were co-transfected with HA-CSN6 and then treated with MG132 (20 μM) for 6 hours. The cell lysates were pulled down with anti-Flag M2 agarose beads and immunoblotted with the indicated antibodies. (b) CSN6 knockdown increased the steady-state expression of FBXW7β. HCT116 and HCT8 cells were infected with the indicated plko-CSN6 lentiviral vectors and were selected with 1.5 μg/mL or 2.5 μg/mL puromycin for 72 hours to eliminate non-infected cells. Afterward, 200 ng/mL doxycycline (DOX) was added to the cells for another 48 hours before harvesting. Total lysates were isolated for immunoblotting analyses with the indicated antibodies. (c) CSN6 overexpression reduced the steady-state expression of FBXW7β. HCT116 and DLD1 cells were stably transfected with control vector (plvx vector) or vector expressing CSN6 (plvx CSN6). Total lysates were isolated for immunoblotting analyses with the indicated antibodies. (d) CSN6 and FBXW7β have direct binding. FBXW7β and FASN also have direct binding. However, CSN6 and FASN have no direct binding. Indicated proteins were produced with TNT Quick Coupled Transcription/Translation systems (Promega, L1170). The indicated proteins were immunoprecipitated with anti-Flag M2 agarose beads or anti-myc beads and immunoblotted with the indicated antibodies. * indicates nonspecific band. (e) CSN6 knockdown increased the expression of FBXW7β with concurrent decreased FASN expression. Dox inducible sh*CSN6* HCT116 cells were treated with or without doxycycline (DOX, 200 μg/mL). Cell proteins were extracted and fractionated by gel-filtration chromatography. The eluated positions of proteins are shown. An equal volume from each chromatographic fraction was analyzed by western blotting with indicated antibodies. (f) CSN6 knockdown reduces the turnover rate of FBXW7β. CSN6-depleted DLD1 cells were constructed as indicated in B. 48 hours after doxycycline induction, cells were split into 60-mm dishes. After another 20 h, cells were treated with cycloheximide (CHX, 100 mg/mL) for indicated time. Total lysates were isolated for immunoblotting analyses with the indicated antibodies (left). The relative abundance of remaining FBXW7β protein was normalized to GAPDH and then normalized to the t = 0 controls (right). (g) CSN6 overexpression increased FBXW7β ubiquitination. HEK293T cells transfected with the indicated plasmids were treated with MG132 (20 μM) for 6 hours before harvesting. The ubiquitinated FASN proteins were pulled down with nickel beads and immunoblotted with an anti-Flag antibody. Ni-NTA: nickel-nitrilotriacetic acid beads, Ubi: ubiquitin. (h) Schematic diagram of the domain mapping of CSN6 and deletion mutants of CSN6 were constructed. (i) The MPN domain of CSN6 is critical for CSN6 binding to FBXW7β. HEK293T cells were transfected with the indicated HA-tagged CSN6 constructs together with Flag-FBXW7β. MG132 was added to the cells 6 hours before harvesting. The cell lysates were pulled down with anti-Flag M2 agarose beads and immunoblotted with indicated antibody. (j) A MPN domain deletion mutant of CSN6 lost its activity in increasing FASN ubiquitination. HEK293T cells were co-transfected with the indicated HA-tagged CSN6 constructs, His-ubiquitin and Flag-FBXW7β. Cells were treated with MG132 (20 μM) for 6 hours before harvest. The ubiquitinated FBXW7β proteins were pulled down using nickel beads and detected with anti-Flag antibody. Ni-NTA: nickel-nitrilotriacetic acid beads, Ubi: ubiquitin. (k) Schematic diagram of the domain mapping of FBXW7β and deletion mutants of FBXW7β were constructed. (l) The F-box domain of FBXW7β was required for FBXW7β binding with Cullin-1. HEK293T cells were transfected with HA-Cullin1 together with the indicated Flag-tagged FBXW7β constructs and then treated with MG132 (20 μM) for 6 hours before harvesting. The cell lysates were pulled down with anti-Flag M2 agarose beads and immunoblotted with the indicated antibodies.

Figure. S4.


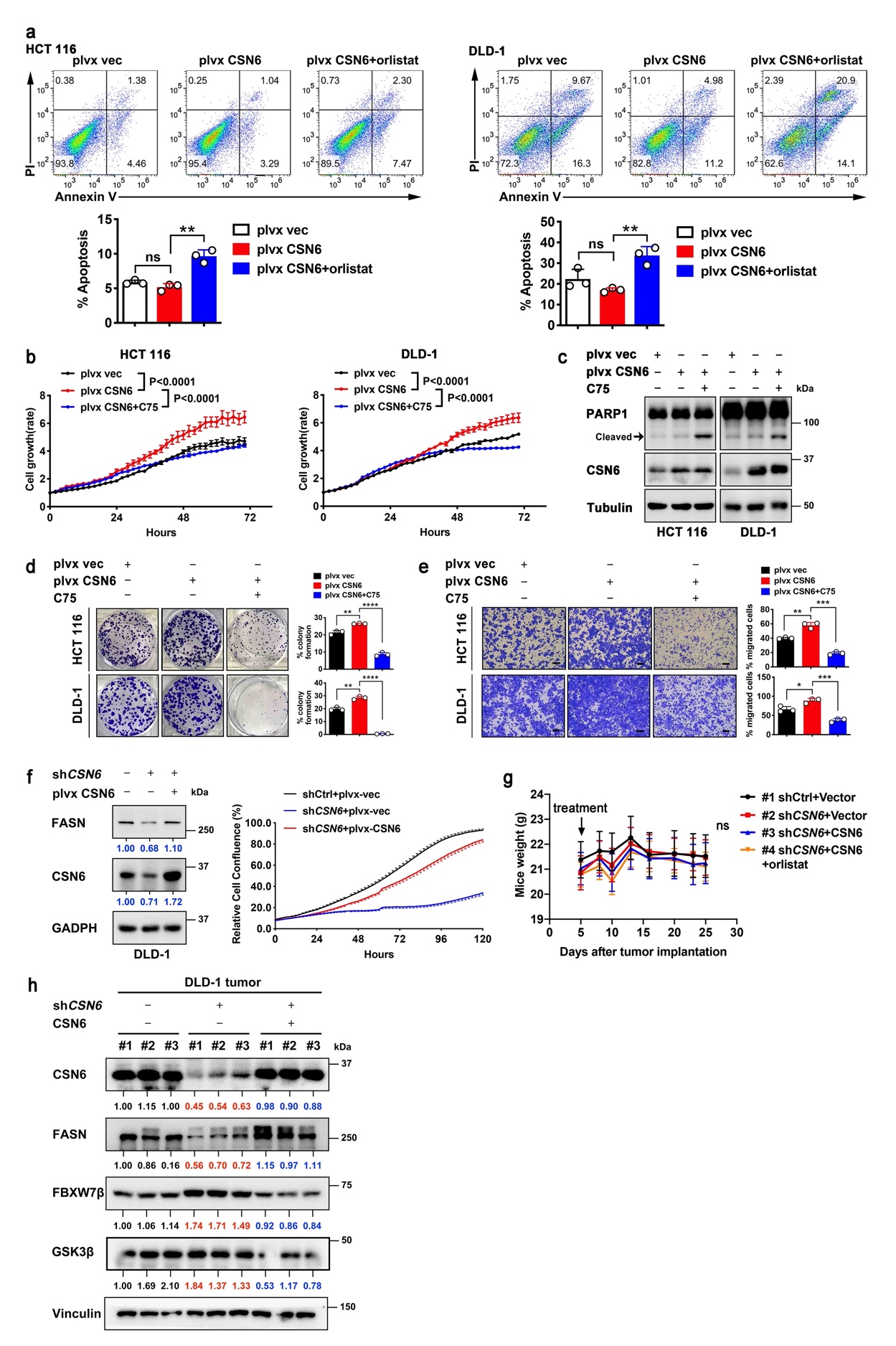


**Figure. S4. Another FASN inhibitor C75 inhibits CSN6-mediated tumor growth in CRC.** (a) Cell apoptosis assay for HCT116 cells and DLD1 cells stably transfected with indicated plasmids and then treated with vehicle (DMSO) or orlistat (8 μM). Representative data of triplicate experiments are shown. Statistical significance of result was tested using one-way ANOVA. Data are represented as mean ± SD, ns not significant, ***P* < 0.01. (b) C75 treatment group demonstrated a robust inhibition in cell proliferation. Cell growth assay generated from HCT116 and DLD1 cells stably transfected with a control vector (plvx vec) or a vector expressing CSN6 (plvx-CSN6) and then treated with vehicle (DMSO) or C75 (8 μM). Statistical significance of result was tested using Two-way ANOVA. (c) C75 treatment induced PARP cleavage. Immunoblot analysis of apoptosis marker Cleaved PARP1 in HCT116 and DLD1 cells stably transfected with a control vector (plvx vec) or a vector expressing CSN6 (plvx-CSN6) and then treated with vehicle (DMSO) or C75 (8 μM) for 3 days. Arrow denotes molecular weight of Cleaved PARP1. (d-e) C75 treatment inhibited colony formation and migration capacity. Colony-formation assays (d) and migration assays (e) generated from HCT116 and DLD1 cells stably transfected with a control vector (plvx vec) or a vector expressing CSN6 (plvx-CSN6) and then treated with vehicle (DMSO) or C75 (8 μM). Representative data of triplicate experiments are shown. Scale bar, 200 μm. Statistical significance of result was tested using One-way ANOVA. Data are represented as mean ± SD, **P* <0.05; ***P* < 0.01; ****P* <0.001; *****P* <0.0001. (f) CSN6 (CSN6 shRNA resistant)-reexpression rescued cell growth inhibition caused by CSN6 KD. The expressions of CSN6 and FASN were tested in above cells (left). Cell growth assay generated from DLD1 cells infected with indicated lentivirus (right). (g) Body weight was measured in mice generated in 6f (*n*=5 mice per group). Statistical significance of result was tested using Two-way ANOVA, ns not significant. (h) CSN6 KD tumors demonstrate decreased FASN protein levels based on immunoblotting. Western blot analysis of indicated proteins in DLD1 tumors generated from 6f. Numbers represent individual tumors from separate mice. Vinculin was used as a loading control.

Figure. S5.


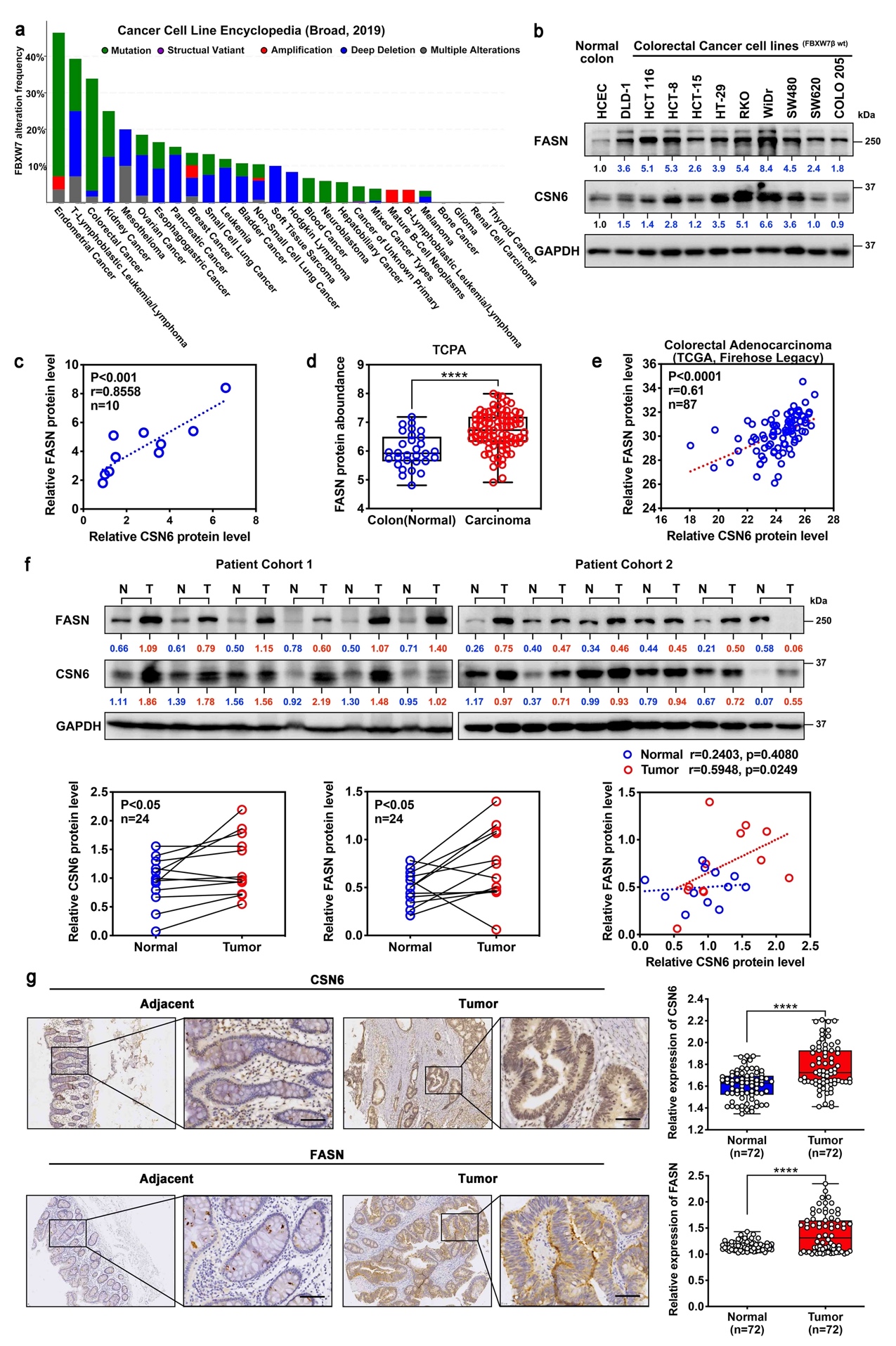


**Figure. S5.** **The Cancer Proteome Atlas (TCPA) and TMA analysis demonstrated that the FASN expression level was higher in cancer tissue than in normal tissue.** (a) FBXW7 is highly mutated in colorectal cancer cell lines. Genetic alterations of FBXW7 from Cancer Cell Line Encyclopedia were indicated (<http://www.cbioportal.org/study/summary?id=ccle_broad_2019>). (b-c) Expression level of CSN6 and FASN was positively correlated in colorectal cancer cell lines. Immunoblot analysis (b) of FASN and CSN6 protein levels in one normal colon cell line (HCEC) and 10 selected FBXW7β-wildtype (wt) colorectal cancer cell lines were shown. Relative protein expression level (normalized to GAPDH) was quantitated and shown below each FASN and CSN6 band. Positive correlation between CSN6 and FASN was shown (c). Each circle is an individual cell line. Spearman’s correlation coefficient was indicated. (d) FASN expression level was higher in cancer tissue than in normal tissue. FASN protein levels were assessed in CRC patients from The Cancer Proteome Atlas (TCPA, <https://tcpaportal.org/tcpa/>). The horizontal lines represent the medians; the boxes represent the interquartile range, and the whiskers represent the 5th and 95th percentiles. Statistical significance of result was tested using two-sided Student’s t test. *****P* < 0.0001. (e) FASN protein levels were positively correlated with the levels of CSN6. FASN and CSN6 protein levels were assessed in CRC patients from Clinical Proteomic Tumor Analysis Consortium database (CPTAC; <https://proteomics.cancer.gov/programs/cptac>). Each circle is an individual sample. Spearman’s correlation coefficient was indicated. (f) The expression of CSN6 and FASN was both elevated in tumor (T) tissue samples from 12 CRC patients when compared with adjacent normal (N) tissues by immunoblot analysis. Relative protein quantitation (normalized to GAPDH) was shown. Relative protein expression of CSN6 or FASN in paired samples of CRC and normal tissue was presented. Statistical significance of result was tested using Pair t test (left and middle). There was a positive correlation between CSN6 and FASN protein levels from tumor samples. Each circle is an individual sample. Spearman’s correlation coefficient was indicated (right). (g) Representative IHC staining for CSN6 and FASN in 72 paired human colon cancer and adjacent normal colon tissue from CRC TMAs (left). Scale bar, 50 μm. Comparison between CSN6 and FASN was shown (right). The horizontal lines represent the medians; the boxes represent the interquartile range, and the whiskers represent the 5th and 95th percentiles. Statistical significance of result was tested using Pair t test. *****P* < 0.0001.

Figure. S6.


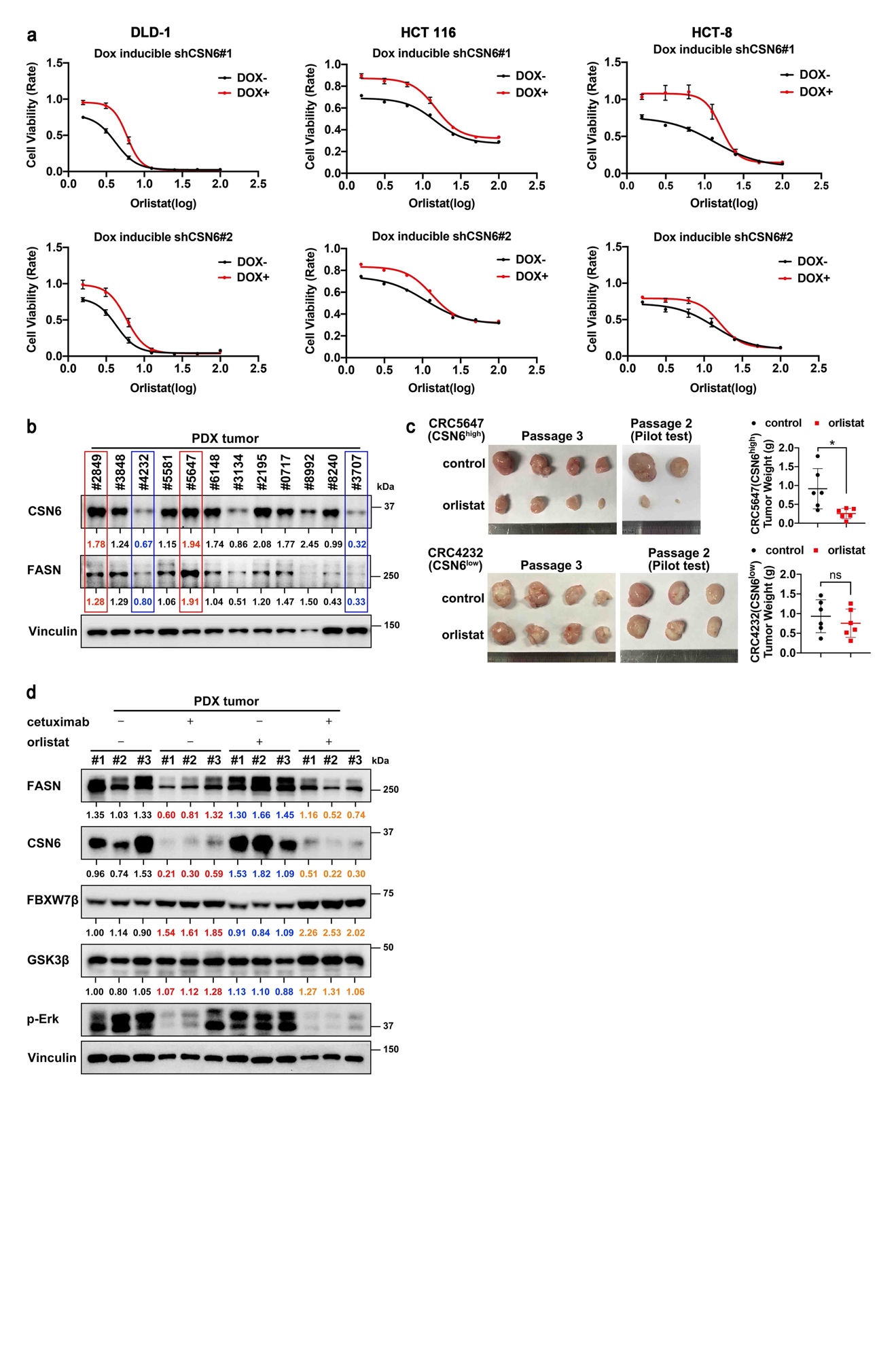


**Figure. S6. CSN6 KD cells are resistant to the growth inhibitory effect of orlistat.** (a) CSN6 knockdown led to an increase in the orlistat IC50. Dox inducible sh*CSN6*#1 and #2 DLD1, HCT116 and HCT8 cells pretreated with or without doxycycline (200 μg/mL) for 48 hours and then treated with increasing doses of orlistat. 72 hours later, cell viability was measured using CCK8 assay. (b) Immunoblot analysis of FASN and CSN6 protein levels in 12 indicated patient-derived xenografts (PDXs) tumor. Relative protein quantitation (normalized to Vinculin) is shown below each FASN and CSN6 band. Red boxes are selected patients with CSN6-overexpressing colon cancer. Blue boxes are selected patients with CSN6-low colon cancer. (c) Representative CSN6 high PDX was shown to be sensitive to orlistat treatment. CSN6 low PDX was shown to be insensitive to the growth inhibitory effect of orlistat. The resulting tumors generated in 7f were resected and photographed at the end of the assay (passage 3), and the pilot test (passage 2) of indicated PDX tumors were also shown. Tumor weight of each group was measured. Statistical significance of result was tested using two-sided Student’s t test. Data are represented as mean ± SD, ns, not significant; **P* < 0.05. (d) Cetuximab-treated tumors demonstrated decreased CSN6 and FASN expression based on immunoblotting. Western blot analysis of indicated proteins in PDX tumors generated from 8f. Number represents individual tumors from mice. Vinculin was used as a loading control.

Figure. S7.


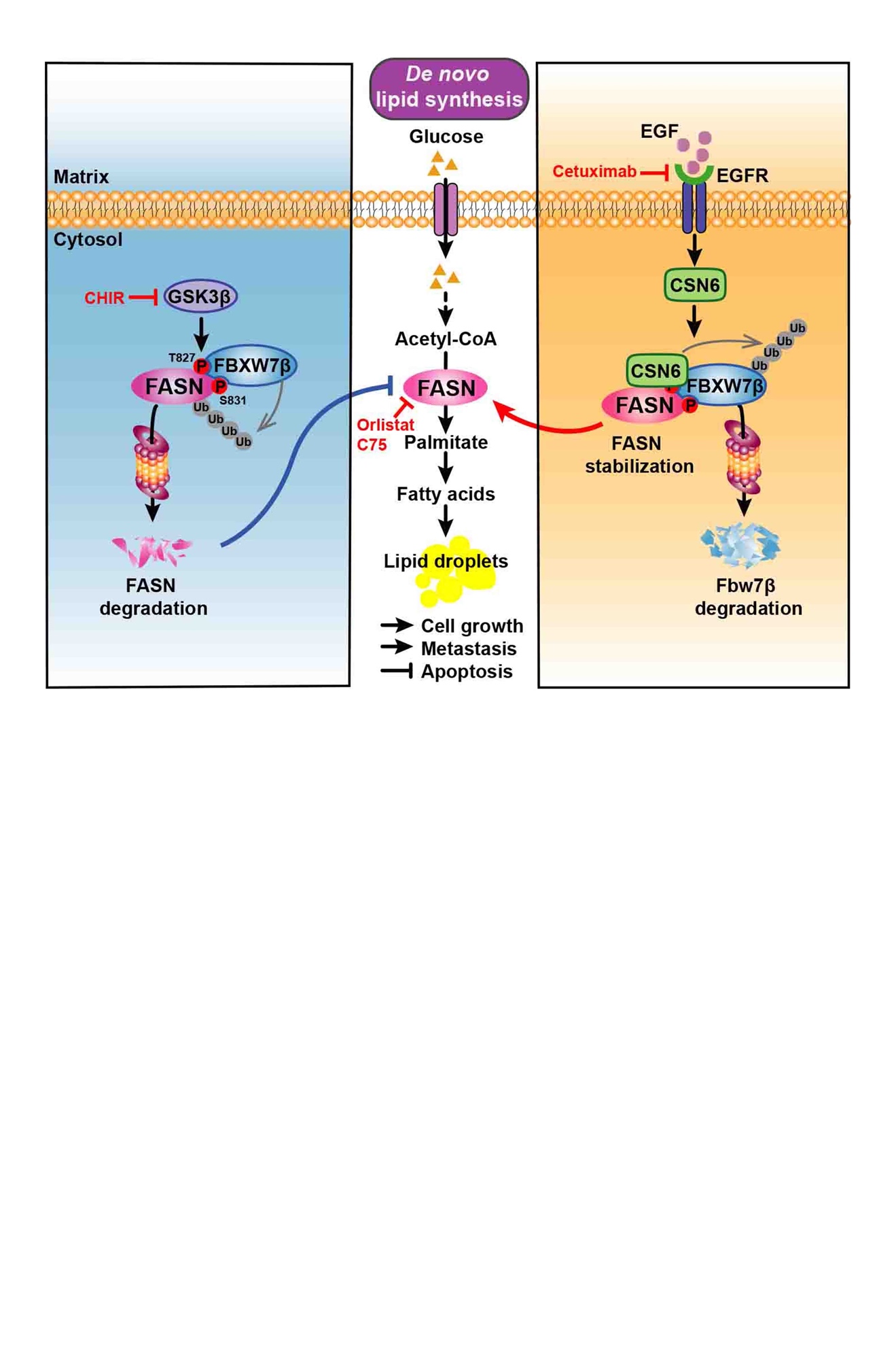


**Figure. S7. Schematic summary of CSN6’s role in modulating FASN stability via E3 ligase FBXW7β.** Schematic summary. Model of CSN6’s role in modulating FASN stability and tumorigenesis. Upon phosphorylation of FASN by the Akt-GSK3β axis, FASN is recognized and degraded by E3 ligase FBXW7β. However, CSN6 associates with FBXW7β, and antagonizes FBXW7β’s activity by enhancing FBXW7β autoubiquitination and degradation, which in turn prevents FBXW7β-mediated FASN ubiquitination and degradation, thereby regulating lipogenesis positively.

Table S1 The top 30 interacting proteins of CSN6

| **Gene Symbol** | **PepCount** | **UniquePepCount** | **MW** |
| --- | --- | --- | --- |
| COP9 constitutive photomorphogenic-like protein subunit 4 isoform 1 (Fragment) | 21 | 17 | 46268.28 |
| COP9 signalosome complex subunit 4 | 21 | 17 | 46268.28 |
| COP9 constitutive photomorphogenic homolog subunit 2 variant (Fragment) | 20 | 16 | 51569.07 |
| COP9 signalosome complex subunit 2 | 20 | 16 | 51596.1 |
| COP9 constitutive photomorphogenic homolog subunit 2 variant (Fragment) | 20 | 16 | 52532.14 |
| COP9 constitutive photomorphogenic-like protein subunit 4 isoform 1 | 11 | 8 | 36163.09 |
| Homo sapiens COP9 subunit 6 (MOV34 homolog, 34 kD) (COPS6) | 11 | 8 | 33575.25 |
| COP9 signalosome complex subunit 6 | 11 | 8 | 36063.95 |
| Cullin-4A | 11 | 11 | 87679.25 |
| Cullin 4B | 11 | 11 | 102754.48 |
| Cullin-4B | 11 | 11 | 103980.47 |
| COP9 signalosome complex subunit 3 | 10 | 8 | 47872.64 |
| COP9 constitutive photomorphogenic homolog subunit 5 (Arabidopsis) | 8 | 6 | 37578.39 |
| COP9 signalosome complex subunit 5 | 8 | 6 | 37578.39 |
| Fatty acid synthase | 7 | 7 | 273423.4 |
| Fatty acid synthase | 7 | 7 | 273195.16 |
| COP9 signalosome complex subunit 7a (Fragment) | 7 | 6 | 23639.79 |
| COP9 signalosome complex subunit 7a | 7 | 6 | 28966.02 |
| COPS7A protein | 7 | 6 | 27317.04 |
| COP9 signalosome complex subunit 7a | 7 | 6 | 30276.24 |
| COP9 signalosome complex subunit 8 | 7 | 5 | 23225.35 |
| COP9 constitutive photomorphogenic homolog subunit 8 (Arabidopsis) | 7 | 5 | 23225.35 |
| highly similar to Homo sapiens COP9 homolog (COP9) | 7 | 5 | 23197.3 |
| COP9 signalosome complex subunit 8 | 7 | 5 | 19344 |
| Cullin-2 | 6 | 6 | 88504.85 |
| Testis secretory sperm-binding protein | 6 | 6 | 86982.1 |
| Cullin-2 | 6 | 6 | 82357.72 |
| Cullin-2 | 6 | 6 | 86982.1 |
| COP9 signalosome complex subunit 7b | 4 | 4 | 29621.62 |

MW, molecular weight.

Table S2 FBXW7β gene alteration status in colorectal cancer cell line

| **Sample ID** | **FBXW7: MUT** | **FBXW7: AMP** | **FBXW7: HOMDEL** | **FBXW7: FUSION** |
| --- | --- | --- | --- | --- |
| SW1116 | H380Y | no alteration | no alteration | no alteration |
| SNUC2A | G499W | no alteration | no alteration | no alteration |
| SNU1197 | R399Q (driver) | no alteration | no alteration | no alteration |
| HT115 | R385C (driver) | no alteration | no alteration | no alteration |
| SNUC5 | S588Vfs*39 (driver) | no alteration | no alteration | no alteration |
| SNUC4 | R393Kfs*4 (driver) | no alteration | no alteration | no alteration |
| SW1463 | R399Q (driver) | no alteration | no alteration | no alteration |
| CCK81 | R385C (driver) | no alteration | no alteration | no alteration |
| SW837 | L323Ffs*34 (driver) | HOMDEL (driver) | HOMDEL (driver) | no alteration |
| GP2D | H500R | no alteration | no alteration | no alteration |
| SNU81 | R399Q (driver), D551Y | no alteration | no alteration | no alteration |
| SNU175 | R385C (driver), A546V, E449D | no alteration | no alteration | no alteration |
| SNU407 | R385C (driver) | no alteration | no alteration | no alteration |
| RCM1 | R425C (driver) | no alteration | no alteration | no alteration |
| LS411N | R425H (driver) | no alteration | no alteration | no alteration |
| LOVO | R425C (driver) | no alteration | no alteration | no alteration |
| SW48 | S588Vfs*39 (driver) | no alteration | no alteration | no alteration |
| CL14 | no alteration | HOMDEL (driver) | HOMDEL (driver) | no alteration |
| CL11 | R399Q (driver) | no alteration | no alteration | no alteration |
| SNUC2B | G499W | not profiled | not profiled | no alteration |
| GP5D | H500R | not profiled | not profiled | no alteration |
| HCC2998 | R609W (driver) | not profiled | not profiled | no alteration |
| COLO205 | no alteration | no alteration | no alteration | no alteration |
| OUMS23 | no alteration | no alteration | no alteration | no alteration |
| NCIH716 | no alteration | no alteration | no alteration | no alteration |
| SW480 | no alteration | no alteration | no alteration | no alteration |
| NCIH508 | no alteration | no alteration | no alteration | no alteration |
| LS123 | no alteration | no alteration | no alteration | no alteration |
| CW2 | no alteration | no alteration | no alteration | no alteration |
| SNU61 | no alteration | no alteration | no alteration | no alteration |
| SNU1033 | no alteration | no alteration | no alteration | no alteration |
| HCT15 | no alteration | no alteration | no alteration | no alteration |
| COLO201 | no alteration | not profiled | not profiled | no alteration |
| C2BBE1 | no alteration | no alteration | no alteration | no alteration |
| LS513 | no alteration | no alteration | no alteration | no alteration |
| HT55 | no alteration | no alteration | no alteration | no alteration |
| MDST8 | no alteration | no alteration | no alteration | no alteration |
| SKCO1 | no alteration | no alteration | no alteration | no alteration |
| HCT8 | not profiled | not profiled | not profiled | not profiled |
| SNU283 | no alteration | no alteration | no alteration | no alteration |
| SNU503 | no alteration | no alteration | no alteration | no alteration |
| HCC56 | no alteration | no alteration | no alteration | no alteration |
| RKO | no alteration | no alteration | no alteration | no alteration |
| SNUC1 | no alteration | no alteration | no alteration | no alteration |
| SW620 | no alteration | no alteration | no alteration | no alteration |
| HCT116 | no alteration | no alteration | no alteration | no alteration |
| DLD1 | not profiled | no alteration | no alteration | not profiled |
| LS1034 | no alteration | no alteration | no alteration | no alteration |
| LS180 | no alteration | no alteration | no alteration | no alteration |
| SW403 | no alteration | no alteration | no alteration | no alteration |
| NCIH747 | no alteration | no alteration | no alteration | no alteration |
| SW1417 | no alteration | no alteration | no alteration | no alteration |
| SW948 | no alteration | no alteration | no alteration | no alteration |
| HT29 | no alteration | no alteration | no alteration | no alteration |
| KM12 | no alteration | no alteration | no alteration | no alteration |
| T84 | no alteration | no alteration | no alteration | no alteration |
| CL40 | no alteration | no alteration | no alteration | no alteration |
| COLO678 | no alteration | no alteration | no alteration | no alteration |
| COLO320 | no alteration | no alteration | no alteration | no alteration |
| CL34 | no alteration | no alteration | no alteration | no alteration |
| HRT18 | no alteration | no alteration | no alteration | no alteration |
| CACO2 | no alteration | not profiled | not profiled | no alteration |
| CAR1 | no alteration | not profiled | not profiled | no alteration |
| NCIH630 | no alteration | not profiled | not profiled | no alteration |
| SW626 | not profiled | not profiled | not profiled | not profiled |
| DIFI | no alteration | not profiled | not profiled | no alteration |
| LIM1215 | no alteration | not profiled | not profiled | no alteration |
| GEO | no alteration | not profiled | not profiled | no alteration |
| HS675T | not profiled | no alteration | no alteration | not profiled |
| HS698T | not profiled | no alteration | no alteration | not profiled |
| C10 | no alteration | not profiled | not profiled | no alteration |
| C125PM | no alteration | not profiled | not profiled | no alteration |
| C75 | no alteration | not profiled | not profiled | no alteration |
| C80 | no alteration | not profiled | not profiled | no alteration |
| C84 | no alteration | not profiled | not profiled | no alteration |
| C99 | no alteration | not profiled | not profiled | no alteration |
| COLO320HSR | not profiled | not profiled | not profiled | not profiled |

Table S3 Correlation between expression of CSN6 and FASN and clinicopathological features of colorectal cancer patients

| **Variable** | **Low CSN6** | **High CSN6** | **p value** | **Low FASN** | **High FASN** | **p value** |
| --- | --- | --- | --- | --- | --- | --- |
| Gender |  |  | 0.9327 |  |  | 0.2044 |
| Male | 22 (56.4) | 35 (55.6) |  | 30 (67.5) | 27 (50.0) |  |
| Female | 17 (43.6) | 28 (44.4) |  | 18 (37.5) | 27 (50.0) |  |
| Median age |  |  | 0.6677 |  |  | 0.7642 |
| ＜59 years | 7 (18.4) | 13 (22.0) |  | 12 (25.0) | 8 (16.3) |  |
| ≥59 years | 31 (81.6) | 46 (78.0) |  | 36 (75.0) | 41 (83.7) |  |
| Histological grade |  |  | 0.0068 |  |  | 0.0326 |
| Ⅰ-Ⅱ | 4 (10.3) | 3 (4.7) |  | 6 (12.5) | 1 (1.8) |  |
| Ⅱ | 26 (66.7) | 25 (39.1) |  | 27 (56.3) | 24 (43.6) |  |
| Ⅱ-Ⅲ | 5 (12.8) | 28 (43.8) |  | 10 (20.8) | 23 (41.8) |  |
| Ⅲ | 4 (10.3) | 8 (12.5) |  | 5 (10.4) | 7 (12.7) |  |
| pT status |  |  | 0.1975 |  |  | 0.0792 |
| T1 | 1 (2.8) | 0 (0) |  | 1 (2.2) | 0 (0) |  |
| T2 | 2 (5.6) | 2 (3.2) |  | 2 (4.3) | 2 (3.8) |  |
| T3 | 31 (86.1) | 50 (79.4) |  | 41 (89.1) | 40 (75.5) |  |
| T4 | 2 (5.6) | 11 (17.5) |  | 2 (4.3) | 11 (20.8) |  |
| pN status |  |  | 0.0798 |  |  | 0.1280 |
| N0 | 29 (76.3) | 34 (54.0) |  | 34 (72.3) | 29 (53.7) |  |
| N1 | 6 (15.8) | 20 (31.7) |  | 8 (17.0) | 18 (33.3) |  |
| N2 | 3 (7.9) | 9 (14.3) |  | 5 (10.6) | 7 (13.0) |  |
| pM status |  |  | 0.1700 |  |  | 0.1881 |
| M0 | 39 (100) | 61 (95.3) |  | 48 (100) | 52 (94.6) |  |
| M1 | 0 (0) | 3 (4.7) |  | 0 (0) | 3 (5.5) |  |
| Clinical stage |  |  | 0.0834 |  |  | 0.1330 |
| Ⅰ | 3 (7.7) | 1 (1.6) |  | 3 (6.3) | 1 (1.9) |  |
| Ⅱ | 26 (66.7) | 33 (52.4) |  | 31 (64.6) | 28 (51.9) |  |
| Ⅲ | 10 (25.6) | 26 (41.3) |  | 14 (29.2) | 22 (40.7) |  |
| Ⅳ | 0 (0) | 3 (4.8) |  | 0 (0) | 3 (5.6) |  |

p values were calculated in SPSS25.0 using a chi-square test. p values＜0.05 were considered to indicate statistical significance.

Table S4 Univariate and multivariate analysis of different prognostic parameters for colorectal cancer patients

| **Variable** | **Univariate analysis** | | **Multivariate analysis** | |
| --- | --- | --- | --- | --- |
|  | **HR (95%CI)*^a^*** | **p value*^b^*** | **HR (95%CI)*^a^*** | **p value*^b^*** |
| cohort (*n*=103) |  |  |  |  |
| Gender (male versus female) | 0.9 (0.5-1.5) | 0.669 | 0.5 (0.3-1.0) | 0.043 |
| Age (<59 years versus >59 years) | 2.1 (1.0-4.5) | 0.045 | 3.2 (1.4-7.5) | 0.008 |
| Histological grade (I-II, Ⅱ, Ⅱ-Ⅲ or Ⅲ) | 1.4 (1.0-1.9) | 0.045 | 1.2 (0.8-1.9) | 0.316 |
| pT status (T1, T2, T3 or T4) | 2.0 (1.1-3.6) | 0.016 | 1.8 (0.9-3.7) | 0.096 |
| pN status (N0, N1 or N2) | 2.3 (1.6-3.2) | <0.001 | 3.8 (1.6-8.6) | 0.002 |
| pM status (M0 versus M1) | 14.0 (3.9-49.8) | <0.001 | 14.1 (1.4-140.0) | 0.024 |
| Clinical stage (I, II, III or IV) | 3.0 (1.9-4.8) | <0.001 | 0.7 (0.2-2.3) | 0.596 |
| CSN6-FASN expression |  |  |  |  |
| Low CSN6-Low FASN |  | 0.003 |  | 0.139 |
| Low CSN6-High FASN | 1.5 (0.6-4.0) | 0.375 | 1.6 (0.6-4.6) | 0.382 |
| High CSN6-Low FASN | 1.8 (0.8-4.2) | 0.169 | 0.9 (0.3-2.4) | 0.813 |
| High CSN6-High FASN | 3.4 (1.7-6.7) | <0.001 | 2.1 (0.9-4.7) | 0.086 |

*^a^*Hazard ratios (HRs) and 95% confidence intervals (CIs) were calculated using univariate or multivariate Cox proportional hazards regression in SPSS 25.0.

*^b^*p values were calculated using univariate or multivariate Cox proportional hazards regression in SPSS 25.0. p values ＜0.05 were considered to indicate statistical significance.

Table S5 Sequences of the mutagenic primers

| **Target** | **Sequences** |
| --- | --- |
| FBXW7β-R425C | S 5’-AGCAGTCtgcTGTGTTCAATATGATGGCAGGAG-3’ |
|  | AS 5’-GAACACAgcaGACTGCTGCAACATGACCCATC-3’ |
| FBXW7β-R425H | S 5’-AGCAGTCcacTGTGTTCAATATGATGGCAGGAG-3’ |
|  | AS 5’-GAACACAgtgGACTGCTGCAACATGACCCATC-3’ |
| FBXW7β-R385C | S 5’-CACTGTGtgtTGTATGCATCTTCATGAAAAAAGAGTT-3’ |
|  | AS 5’-GCATACAacaCACAGTGGAAGTATGCCCATATAAG-3’ |
| FBXW7β-R385H | S 5’-CACTGTGcatTGTATGCATCTTCATGAAAAAAGAGTT-3’ |
|  | AS 5’-GCATACAatgCACAGTGGAAGTATGCCCATATAAG-3’ |
| FBXW7β-R399Q | S 5’-CGGTTCTcaaGATGCCACTCTTAGGGTTTGGG-3’ |
|  | AS 5’-TGGCATCttgAGAACCGCTAACAACTCTTTTTTCA-3’ |
| FASN-T827A/S831A | S 5’-CGAGGAgctCCCCTCATCgccCCACTCATCAAGTGG-3’ |
|  | AS 5’-GATGAGGGGagcTCCTCGGGGAGCTGGGAACTCCAC-3’ |
| FASN-T827D/S831D | S 5’-CGAGGAgatCCCCTCATCgacCCACTCATCAAGTGG-3’ |
|  | AS 5’-GATGAGGGGatcTCCTCGGGGAGCTGGGAACTCCAC-3’ |

S, sense; AS, antisense.

Table S6 Primers for qRT-PCR

| **Target** | **Sequences** |
| --- | --- |
| *CSN6* | S 5’-TCATCGAGAGCCCCCTCTTT-3’ |
|  | AS 5’-CCAATGCGTTCCGCTTCCT-3’ |
| *FBXW7α* | S 5’-CGGAGTCTCCCAAACCTGAC-3’ |
|  | AS 5’-GGCGCGGTACTCCTCTTTT-3’ |
| *FBXW7β* | S 5’-ATGTGTGTCCCGAGAAGCG-3’ |
|  | AS 5’-GATTAGGGAGCAGAACCGGC-3’ |
| *FBXW7γ* | S 5’-GGTCAGGACATTTGGTAGGG-3’ |
|  | AS 5’-GACAAAAAGGGAGGCCTTGG-3’ |
| *FASN* | S 5’-AACCGGCTCTCCTTCTTCTT-3’ |
|  | AS 5’-TTGGGCTTCAGCAGGACATT-3’ |
| *β-actin* | S 5’-GCCGACAGGATGCAGAAGGAGATCA-3’ |
|  | AS 5’-AAGCATTTGCGGTGGACGATGGA-3’ |

S, sense; AS, antisense.
